# Cytosolic iron–sulfur protein assembly system identifies clients by a C-terminal tripeptide

**DOI:** 10.1073/pnas.2311057120

**Published:** 2023-10-26

**Authors:** Melissa D. Marquez, Carina Greth, Anastasiya Buzuk, Yaxi Liu, Catharina M. Blinn, Simone Beller, Laura Leiskau, Anthony Hushka, Kassandra Wu, Kübra Nur, Daili J. A. Netz, Deborah L. Perlstein, Antonio J. Pierik

**Affiliations:** ^a^Department of Chemistry, Boston University, Boston, MA 02215; ^b^Department of Chemistry, University of Kaiserslautern-Landau, Kaiserslautern 67663, Germany

**Keywords:** iron–sulfur, metallocofactor, cytosolic iron-sulfur protein assembly (CIA) machinery

## Abstract

The eukaryotic cytosolic Fe–S protein assembly (CIA) machinery inserts iron–sulfur (Fe–S) clusters into cytosolic and nuclear proteins. In the final maturation step, the Fe–S cluster is transferred to the apo-proteins by the CIA-targeting complex (CTC). However, the molecular recognition determinants of client proteins are unknown. We show that a conserved [LIM]-[DES]-[WF]-COO^–^ tripeptide is present at the C-terminus of more than a quarter of clients or their adaptors. When present, this targeting complex recognition (TCR) motif is necessary and sufficient for binding to the CTC in vitro and for directing Fe–S cluster delivery in vivo. Remarkably, fusion of this TCR signal enables engineering of cluster maturation on a nonnative protein via recruitment of the CIA machinery. Our study advances our understanding of Fe–S protein maturation and paves the way for bioengineering novel pathways containing Fe–S enzymes.

## Significance

The maturation of eukaryotic cytosolic and nuclear iron–sulfur (Fe–S) proteins is mediated by the cytosolic iron–sulfur protein assembly (CIA) pathway. Unlike other metalloprotein maturation systems with a one-to-one correspondence between the metallochaperone and its apo-client, CIA must identify >30 clients, raising the question of how specificity of cofactor delivery is encoded. The discovery that a tripeptide motif at the C-terminal tail of CIA clients is necessary and sufficient to direct Fe–S cluster delivery from the CIA system not only provides fundamental insights into biochemistry of this vital process but it also unlocks the potential for bioengineering Fe–S cluster delivery to nonnative enzymes, a bottleneck that often frustrates the use of these versatile metalloproteins for synthetic biology applications.

Iron–sulfur (Fe–S) proteins catalyze transformations vital for life-sustaining processes including photosynthesis, respiration, and nitrogen fixation. Although Fe–S cofactors are readily inserted into apo-proteins in vitro, their biosynthesis requires dedicated pathways that assemble the metalloclusters and shepherd these cofactors to apo-protein clients (1). Cluster biogenesis is essential for iron homeostasis, energy metabolism, genome stability, and other critical cellular processes impacting human health (2–5). The diversity of transformations catalyzed by Fe–S enzymes also makes them attractive candidates for bioengineering of novel pathways, however, inefficient cluster delivery to nonnative proteins often frustrates pharmaceutical and biofuel applications (*SI Appendix*, Table S1) (6, 7). Although progress toward understanding the apo-client recognition has been made in some systems (7–9), there are no plug-and-play solutions to overcome bottlenecks associated with inefficient metallocofactor maturation.

**Fig. 1.**
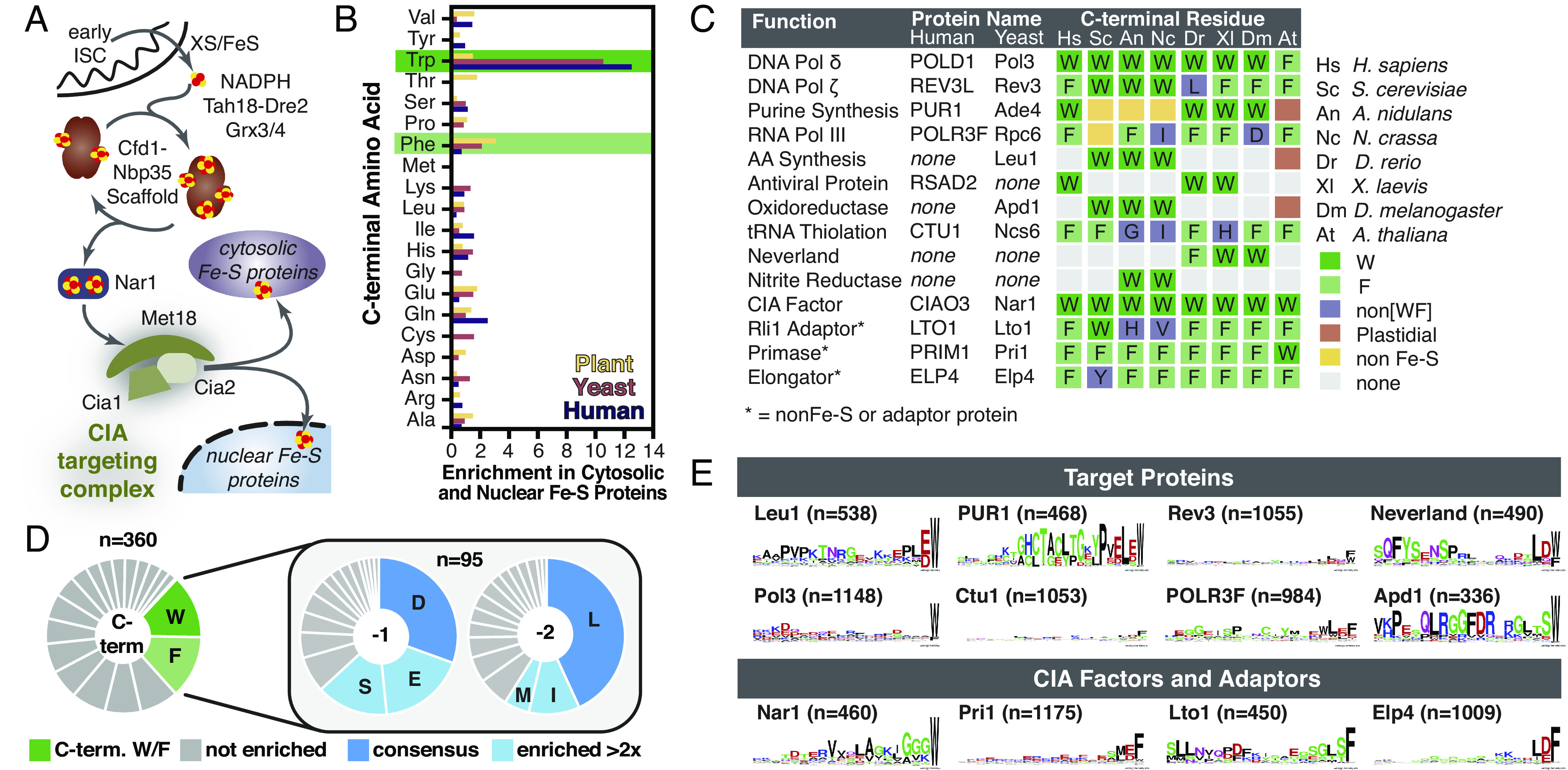
A conserved [LIM]-[DES]-[WF]-COO^-^ tripeptide is found at the C-terminus of CIA clients. (*A*) Overview of the CIA pathway (yeast nomenclature). In the final step, the CTC identifies the CIA clients through direct, or adaptor-mediated, protein–protein interactions and delivers the Fe–S cluster. (*B*) The enrichment of C-terminal amino acids (frequency in dataset/frequency in C-terminal proteome) in cytosolic and nuclear Fe–S proteomes of plants (*Arabidopsis thaliana*, yellow), yeast (*Saccharomyces cerevisiae*, pink), and in humans (*Homo sapiens*, purple). (*C*) The C-terminal residue of Fe–S proteins, CIA factors, and adaptors terminating in W/F in *H. sapiens* (Hs), *S. cerevisiae* (Sc), *Aspergillus nidulans* (An), *Neurospora crassa* (Nc), *Danio rerio* (Dr), *Xenopus laevis* (Xl), *Drosophila melanogaster* (Dm), and *A. thaliana* (At). (*D*) Pie charts showing frequency of amino acids found at the C-terminus (n = 360 sequences), and the -1 (penultimate) and -2 positions (n = 95 sequences, those from the C-terminal dataset terminating in W/F). Amino acids indicated are enriched >twofold (light green or blue) or >threefold (dark green or blue) relative to their frequency in the human proteome. (*E*) WebLogos depicting conservation of C-termini for [4Fe–4S] proteins (Leu1, Pol3, Rev3, POLR3F, PUR1, and Ctu1), [2Fe–2S] proteins (Neverland and Apd1), and the CIA factors/adaptors (Nar1, Lto1, Pri1, and Elp4).

Eukaryotic Fe–S biogenesis is complex because each subcellular compartment houses its own dedicated machinery (1). Cytosolic and nuclear Fe–S protein assembly (CIA) requires several mitochondrial iron–sulfur cluster (ISC) maturation components, including the Nfs1 cysteine desulfurase, and at least 9 additional cytosolic proteins. The ISC machinery synthesizes a molecule containing iron and/or sulfur (XS; Fe–S_int_) (10, 11). Upon export, several protein factors use this molecule for [4Fe–4S] cluster assembly on the Cfd1-Nbp35 CIA scaffold (Fig. 1*A*) (12, 13). Nar1, a 2x[4Fe–4S] protein acts as the Fe–S cluster carrier, associates with the CIA targeting complex (CTC, Fig. 1*A*), and provides the cluster to be inserted into apo-Fe–S targets (14). These targets are recognized by the CTC, a complex comprising Met18/MMS19 (yeast/human nomenclature), Cia1/CIAO1, and Cia2/CIAO2 (15–18).

One major question addressed herein is how the CTC identifies its >30 clients, which include Fe–S proteins essential for DNA replication, transcription, translation, and other cellular processes (19). Proteomics and gene depletion studies have suggested that some clients only require a single CTC component for their maturation [e.g., Viperin (RSAD1) requires only CIAO1], whereas other targets depend on two or all three CTC subunits (20, 21). Additionally, the CIA client Rli1 does not directly bind the CTC but depends on the Yae1-Lto1 adaptor for CIA machinery recruitment (22). The diversity of the CIA proteome, combined with the incomplete catalog of CIA clients requiring adaptors, has made it challenging to unravel the cryptic codes driving substrate recognition by the CTC.

Despite these challenges, some recent studies indicated that some targets might exploit signals at their C-termini to recruit the CIA machinery. The clients, Viperin and Apd1, and the Lto1 adaptor terminate in a tryptophan residue that is critical for in vivo cofactor maturation (22–24). These studies suggest the tantalizing possibility that CIA clients use conserved recognition motifs to recruit the CTC, reminiscent of the recent proposal that a lysine-tyrosine-arginine (LYR) tripeptide motif guides Fe–S cluster delivery from ISC (8). However, experimental support for this proposal is lacking, particularly regarding the number of eukaryotic Fe–S proteins bearing such signals, whether additional other residues in the primary or tertiary structure contribute to recognition and whether these determinants are sufficient for CIA machinery recruitment. Here, we show that >30% of CIA clients or their adaptors have a [LIM]-[DES]-[WF]-COO^-^ tripeptide motif that recruits the CIA machinery and demonstrate using integrative in vitro and in vivo approaches that this targeting complex recognition (TCR) signal is both necessary and sufficient for binding the CTC and delivering Fe–S clusters from the CIA machinery.

## Results

### A C-terminal Aromatic Residue Is Enriched and Conserved in CIA Clients.

By cataloging the C-terminal residue of every Fe–S protein in Fungi (*Saccharomyces cerevisiae*) and Metazoa (*Homo sapiens*) (25), we found that a C-terminal tryptophan residue was uniquely enriched in cytosolic and nuclear Fe–S proteins. Six (24%) of the yeast and five (13%) of the human CIA clients, factors, and adaptors terminated in a tryptophan (Fig. 1*B* and Dataset S1). For comparison, only 1.8% and 1.2% of all yeast and human proteins, respectively, end with a tryptophan. For plant (*Arabidopsis thaliana*) CIA clients (26), the most enriched C-terminal residue was phenylalanine, occurring in seven (17%) of 42 non-glutaredoxin cytosolic and nuclear Fe–S proteins (Fig. 1*B* and Dataset S1). The proportion of TCR-containing CIA clients and adaptors of bacterial origin is 86% and 78% for yeast and human proteins, respectively (Dataset S1). These percentages are lower for proteins lacking a TCR motif (yeast, 60%; humans, 65%). In contrast, a smaller fraction of TCR-containing proteins have an archaeal origin (yeast, 57%; humans, 67%), and the proportion of CIA clients lacking a TCR motif is larger (yeast, 90%; humans 81%). These observations suggest that acquisition of the TCR might have occurred after transfer of bacterial genes to the nucleus (27).

Remarkably, none of the 164 yeast, human, or plant mitochondrial or plastidial Fe–S proteins terminated with a tryptophan or phenylalanine (*SI Appendix*, Fig. S1 and Dataset S1). Similarly, just two (0.8%) of the 248 Fe–S proteins from Bacteria (*E. coli*) and Archaea (*Methanocaldococcus jannaschii*) terminate with tryptophan and ten (4%) with phenylalanine (*SI Appendix*, Fig. S1*D* and Dataset S1) (28–30). Therefore, a C-terminal tryptophan or phenylalanine is a hallmark of CIA clients—the first clear example of a putative CIA-targeting sequence.

Next, 360 CIA clients from 10 model organisms were analyzed to challenge the universality of this hallmark and uncover any additionally conserved features (Fig. 1*C* and Dataset S2). Of the 95 sequences terminating in W or F (26% of 360 sequences), all had conserved amino acids in the penultimate (-1) and antepenultimate (-2) position (Fig. 1*D* and Dataset S2). A negatively charged residue (48%) or a serine (15%) was preferred in the penultimate position, whereas a hydrophobic residue occurs at the antepenultimate position in 59% of the sequences. Sequence alignments and analysis revealed conservation is limited to the -1 and -2 positions (Fig. 1*E* and Dataset S2). We named this tripeptide ([LIM]-[DES]-[WF]-COO^-^) motif the TCR signal, due to its role in the recruitment of the CTC (vide infra).

When CIA clients terminating in the TCR signal were compared to their homologs that are matured by other Fe–S biogenesis machineries, we only found the tripeptide motif to be exclusively conserved in CIA clients. For example, fungal isopropylmalate isomerases (Leu1) are cytosolic [4Fe–4S] proteins with a TCR signal (Fig. 1*E*), but bacterial, archaeal, mitochondrial, and plastidial orthologs are missing the tripeptide (*SI Appendix*, Fig. S2 *A* and *B*). Analogous correspondences occur for other classes of Fe–S proteins, including the CIA factor Nar1; the [4Fe–4S] protein Viperin, Ctu1, and nitrite reductase; and the [2Fe–2S] proteins Apd1, Neverland, and choline monooxygenase (*SI Appendix*, Fig. S2). The length of the TCR-tail varies between ∼3 and 45 amino acid residues (*SI Appendix*, Fig. S2, boxed), suggesting the tail length tunes positioning of different clients. Available proteomic mass spectrometry data and structures of Fe–S proteins isolated from the native host demonstrate that the TCR motif remains attached after maturation (*SI Appendix*, Table S2), in contrast to mitochondrial import signals.

Additionally, some Fe–S clusters play a structural or regulatory role and therefore their cluster binding residues can be lost with evolutionary drift. Remarkably, the TCR signal and Fe–S ligands show strong conservation correlation. For example, many glutamine phospho-ribosyl pyrophosphate amidotransferases, such as PUR1 in humans, are [4Fe–4S] proteins and they terminate with a TCR signal (Fig. 1*E*). In contrast, TCR signals are absent in the plant enzymes, which are plastidial and thus are not matured by CIA, and in their fungal homologs, which lack Fe–S clusters (*SI Appendix*, Fig. S3*A*). RNA polymerases displayed similar coconservation of TCR and Fe–S ligands (*SI Appendix*, Fig. S3*B*). These correlations strongly suggest that the TCR signal is functionally related to CIA.

**Fig. 2.**
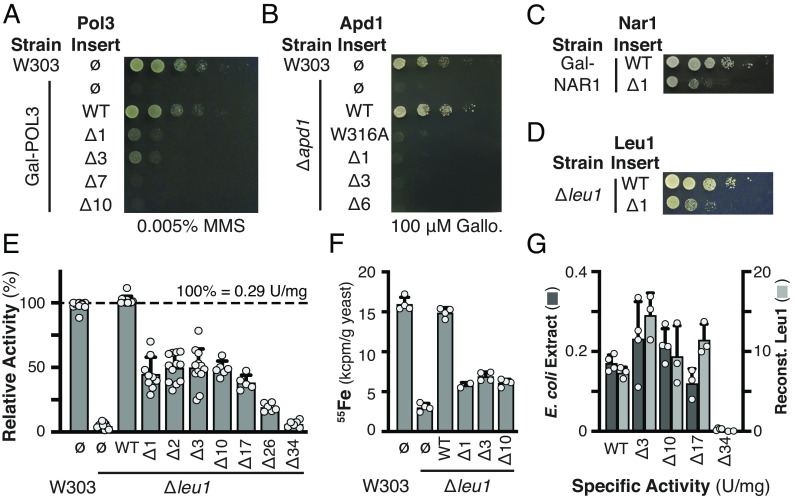
Deletion of the C-terminal tryptophan of CIA clients results in defective Fe–S cluster delivery. (*A*–*C*) The indicated yeast strains, wild-type W303, *apd1* deletion, or Gal-regulable *POL3* or *NAR1*, were transformed with a centromeric plasmid for expression of Pol3 (*A*), Apd1 (*B*), or Nar1 (*C*) from their natural promoters. The Δ indicates the number of C-terminal amino acids that were removed, ø indicates the empty vector control, and WT indicates the full-length wild-type insert. Yeast cells were grown overnight in Glc containing medium, then spotted after serial dilution onto Glc medium with the indicated concentrations of MMS or gallobenzophenone (Gallo.). (*D*) Plasmid-borne Leu1 was expressed from the weak (*RET2*) promoter in a Δ*leu1* strain with a functional *LEU2* allele. Yeast were grown as in *A*–*C*, but in the absence of leucine. (*E* and *F*) Determination of Leu1 enzymatic activity in yeast crude extracts and ^55^Fe incorporation to quantify Fe–S cluster insertion into Leu1. W303 or Δ*leu1* strains harboring the indicated Leu1 variant (expressed from its natural promoter) were grown as in *A*–*C*. For de novo ^55^Fe incorporation, cells were grown in Fe-free medium overnight, followed by incubation with ^55^Fe and immunoprecipitation of Leu1. (*G*) Fe–S cluster insertion by the *E. coli* ISC machinery (cell extract, dark gray) or by chemical reconstitution of Leu1’s Fe–S cluster following purification (light gray) are unaffected by C-terminal truncation up to 17 amino acids.

Next, we truncated the TCR motif at the C-terminus of Pol3, the catalytic subunit of DNA polymerase δ (31). Pol3 is essential for yeast viability upon DNA damage with methylmethane sulfonate (MMS) (Fig. 2*A*). Chromosomal *POL3* was placed under control of a galactose-regulatable promoter (Gal-*POL3*). This strain’s phenotypic growth defect in the presence of MMS and glucose (Glc) was alleviated by episomal Pol3 expression at endogenous levels but not by modified genes encoding truncated Pol3 variants (Fig. 2*A*). In similar experiments, C-terminally truncated variants of Apd1, Nar1, and Leu1 were unable to restore normal growth when expressed at low levels in Gal-regulatable (GAL-*NAR1*) or deletion (Δ*apd1*, Δ*leu1*) strains (Fig. 2 *B*–*D* and *SI Appendix*, Figs. S4 and S5). In all cases, removal or substitution of just the C-terminal tryptophan was sufficient to elicit the growth defect (Fig. 2). Thus, when a TCR motif is present, it is universally required for the Fe–S protein function.

Focusing on Leu1, the benchmark enzyme for quantitative measurements of CIA function (32), centromeric plasmids encoding Leu1 truncations were expressed in a Δ*leu1* strain. Deletion of W779 from the TCR (Leu1-Δ1), and a further truncation of up to 17 amino acids, led to 60% loss of Leu1 activity without decreasing the Leu1 expression level (Fig. 2*E* and *SI Appendix*, Fig. S6). Analysis of de novo ^55^Fe incorporation into Leu1 was similarly diminished by TCR truncation (Fig. 2*F*). We speculate that the remaining activity of Leu1-Δ1 results from a second CTC binding determinant (33). At low expression levels, both the TCR and this second determinant are required for efficient maturation, but increased expression of the Leu1-Δ1 or Nar1-Δ1 variants can drive their complexation with the CTC (Fig. 2 *C* and *D* and *SI Appendix*, Fig. S4). Additional controls demonstrated the decreased activity of Leu1-Δ1 was independent of the transcriptional promoter and terminator sequences (*SI Appendix*, Fig. S7). To rule out the possibility that the Leu1 activity loss results from defective protein folding, the Leu1 truncations were expressed in *E. coli*, which does not encode the CIA machinery. Leu1 activity in *E. coli* crude extracts or following its purification and Fe–S cluster reconstitution was undiminished upon removal of ≤17 amino acids (Fig. 2*G*). These data establish that the Leu1 activity loss upon TCR signal truncation results from a specific defect in cluster delivery from the CIA system.

### The TCR Motif Mediates Interaction with the CIA Targeting Complex.

**Fig. 3.**
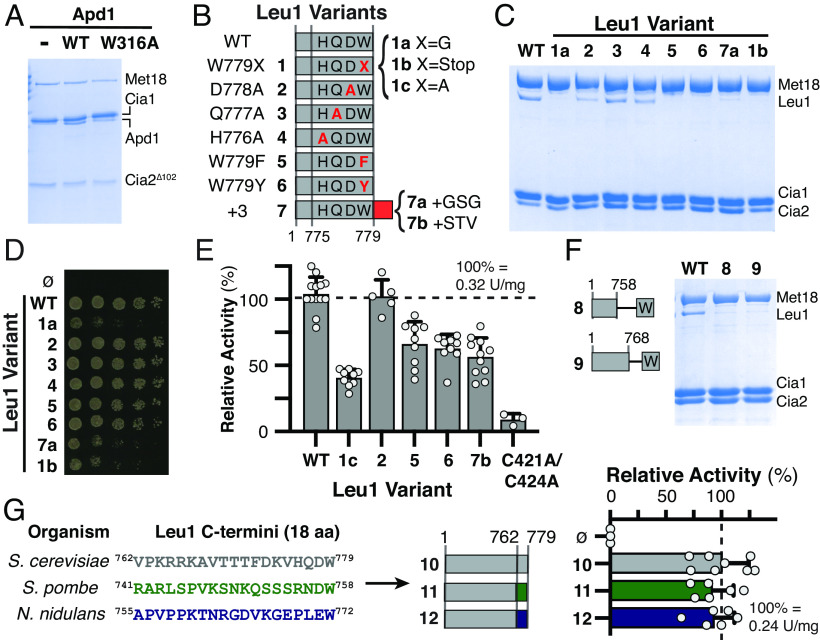
CTC binding in vitro and Fe–S cluster maturation in vivo depend on the C-terminal tryptophan and its carboxy terminus. (*A*) Apd1 (wild-type; WT; W316A variant, or none; -), was mixed with yeast CTC subunits (Met18, Strep-tagged Cia1, and truncated Cia2^Δ102^). Apd1 associated with the CTC was evaluated via SDS-PAGE analysis after Streptactin affinity purification. Due to the overlapping migration of full-length Cia2 and Apd1, the truncated Cia2 construct, Cia2^Δ102^, was utilized (34). (*B*) Variants (red) of WT Leu1 tested in panels (*C*–*E*). Nonbold numbers refer to the positions in the amino acid sequence. (*C*) The Leu1 variants (bolded numbers) were mixed with the yeast CTC (Met18, Cia1, and Strep-tagged Cia2). Leu1 associated with the CTC was evaluated by SDS-PAGE after Streptactin affinity purification. (*D*) Leucine-independent growth of yeast (as in Fig. 2*D*) depends on the C-terminal W of Leu1. (*E*) Determination of Leu1 variant enzymatic activity in yeast cell extract as in Fig. 2*E*. (*F*) Affinity copurification of C-terminal Leu1 truncations as in *B*. (*G*) The last 18 amino acids of *S. cerevisiae* Leu1 (gray, 10) were replaced with Leu1 tails from *S. pombe* (green, 11) or *A. nidulans* (purple, 12). The specific activities of the resulting tail transplant variants were analyzed as in Fig. 2*E*.

The CIA targeting complex (CTC) identifies apo-clients and thus it is the likely TCR signal receptor. To test this hypothesis, we used an affinity copurification assay and sodium dodecylsulfate polyacrylamide gel electrophoresis (SDS-PAGE) to analyze the TCR–CTC interaction, employing the [2Fe–2S] Apd1 and [4Fe–4S] Leu1 client proteins. Both proteins copurified with the CTC, but variants lacking the indole moiety had a defect in CTC binding (Fig. 3 *A*–*C* and *SI Appendix*, Fig. S8). Thus, the TCR tail is critical for CTC interaction in vitro, explaining why TCR truncation leads to a defective Fe–S protein maturation in vivo.

To confirm the functional relevance of the TCR to client protein maturation in vivo, the Leu1 W779 variants were expressed at low levels in a Δ*leu1* strain to evaluate growth in a leucine-deficient medium and at higher levels to quantify Leu1 activity in crude extracts (Fig. 3 *D* and *E* and *SI Appendix*, Fig. S9). The W779A complemented strain grew at a twofold slower rate than strains expressing the wild type protein and exhibited a twofold reduction in Leu1 specific activity (Fig. 3 *D* and *E* and *SI Appendix*, Fig. S9). Western blotting confirmed the decreased activity was not due to differences in protein levels (*SI Appendix*, Fig. S10). Leu1 with W779F/Y substitutions did not pull down with the CTC in the stringent in vitro assay (Fig. 3*C*), but the in vivo functionality of these TCR variants was less severely affected (Fig. 3 *D* and *E* and *SI Appendix*, Fig. S9). Altogether, these results demonstrate that a C-terminal tryptophan is the preferred residue for yeast TCR signals, but TCRs that include a phenylalanine residue can also be recognized.

Next, we extended the C-terminus of Leu1 by three amino acids to determine whether an internal TCR signal retained functionality. This construct was clearly unable to bind the CTC or recruit the CIA machinery in vivo (Fig. 3). Since the D778A, Q777A, and H776A substitutions neither impacted yeast growth nor CTC binding (Fig. 3), our data identify the aromatic side chain and its C-terminal position as the most critical determinants for CTC recruitment.

To examine whether TCR tail length impacts its functionality, we replaced the last 10 or 20 amino acids of Leu1 with a single tryptophan residue. As neither variant copurified with the CTC (Fig. 3*F*), we concluded that either additional amino acids of the tail are critical for recognition by the CTC or the TCR signal has a certain spatial requirement to allow for correct positioning in the CTC. To distinguish between these possibilities, we replaced the last 18 amino acids of the *S. cerevisiae* Leu1 with those from *Schizosaccharomyces pombe* or *Aspergillus nidulans* proteins. Importantly, these three sequences do not share any conserved residues besides the terminal [DE]-W motif (Fig. 3*G*, underlined). Because these tail transplant variants were efficiently matured by the CTC (Fig. 3*G*), we concluded that in addition to the primary sequence determinants, the TCR tail length impacts client positioning for optimal interaction and Fe–S delivery from the CTC.

### The TCR Signal Is Sufficient for Recruitment of CTC to Non-Native Targets.

**Fig. 4.**
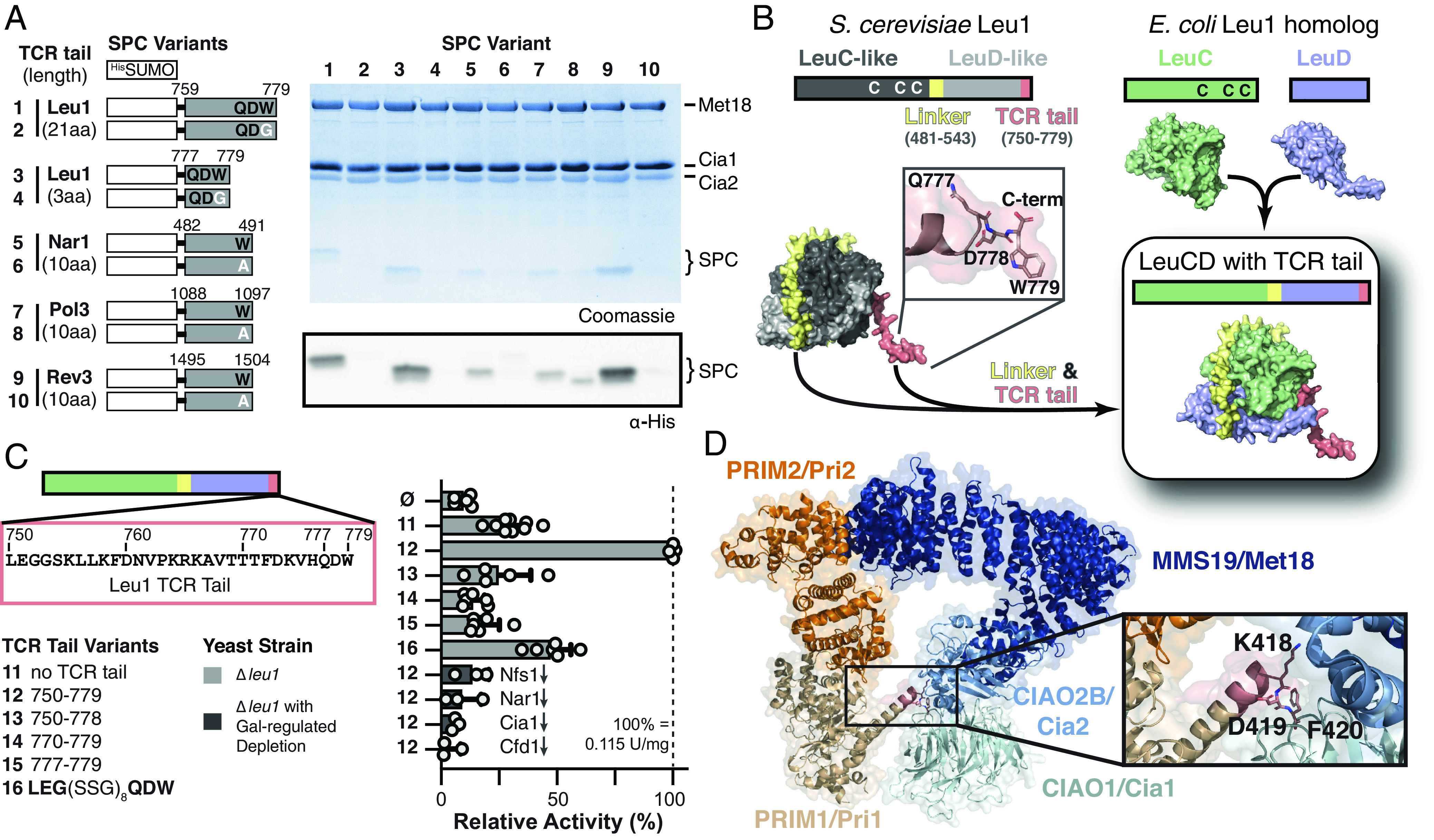
An engineered TCR signal is sufficient to recruit nonnative proteins to the CTC for Fe–S cluster delivery. (*A*) Cartoon of SPC fusions used in copurification, as in Fig. 3*C*. Streptactin elution fractions were analyzed by SDS-PAGE and immunoblotting against the SPC N-terminal His-tag. (*B*) A nonnative Fe–S protein, *E. coli* LeuCD, was engineered for cluster delivery from the CIA machinery. The *E. coli* subunits were fused with a linker (yellow) and the TCR-tail (salmon) of yeast Leu1 was attached. Protein structures are AlphaFold models. (*C*) In vivo cytosolic [4Fe–4S] cluster insertion into engineered *E. coli* LeuCD depends on the TCR signal with an appropriate tail length. Specific activities in cell extracts were determined as in Fig. 2*E*. Each LeuCD variant was expressed in a Δ*leu1* yeast strain (light gray), or a Δ*leu1* strain with galactose-regulatable genes (dark gray). Growth on Glc medium for 40 h led to depletion (↓) of the indicated Fe–S cluster biosynthesis protein. (*D*) Modelling of the PRIM1–PRIM2 (AlphaFold, orange and sand) and CTC (PDB code 6TC0, blue hues) into the cryoelectron microscopy (Cryo-EM) density of the primase–CTC (33). This model shows that the TCR peptide signal (KDF, salmon) interacts with the proposed client binding site on blade 3 of CIAO1.

To eliminate any additional sequence determinants within CIA clients that might contribute to CTC recruitment, we fused various TCR tails to SUMO, a small well-folded protein with an accessible C-terminus. These small ubiquitin-like modifier (SUMO) peptide carriers (SPC) for Leu1_759-779_, Nar1_482-491_, Pol3_1088-1097_, and Rev3_1495-1504_ each bound to the CTC in a tryptophan-dependent manner (Fig. 4*A*). Moreover, the Leu1 tail could be further truncated from 20 to just 3 amino acids (QDW) without disrupting CTC interaction (Fig. 4*A*).

If the TCR motif is sufficient for association with the CTC, then it should be possible to direct Fe–S cluster delivery to a nonnative, heterologous Fe–S protein in yeast. To test this prediction, we focused on the *E. coli* LeuC–LeuD heterodimer, the homolog of yeast Leu1 (Fig. 4*B*). Because the [4Fe–4S] cluster of LeuC–LeuD is biosynthesized by the bacterial ISC system, it does not possess the TCR-extension (*SI Appendix*, Fig. S2 *A* and *B*). The *E. coli leuC* and *leuD* genes were fused with linker derived from yeast Leu1 (yellow, Fig. 4*B*). When the last 30 amino acids of yeast Leu1 were fused to the C-terminus of this LeuCD construct, its enzymatic activity increased >threefold (Fig. 4*C*). To demonstrate this increased activity was due to CIA machinery recruitment, several control experiments were performed. First, we determined that the increased activity of LeuCD–TCR fusion depends on the C-terminal tryptophan (Fig. 4*C*). Second, fractionation of yeast demonstrated LeuCD activity was cytosolic, not mitochondrial where ISC is functional (*SI Appendix*, Fig. S11). Third, GAL-regulatable expression of Fe–S assembly factors in Δ*leu1* strains confirmed the increased activity depends on the CIA machinery and the early ISC machinery providing XS/[Fe–S]_int_ (Fig. 4*C*).

To identify the minimal length required to position LeuCD for cluster delivery, we shortened the TCR tail to 3 or 10 amino acids, but these variants were unable to guide Fe–S maturation (Fig. 4*C*). However, when Leu1 tail residues 753 to 776 were replaced with (SSG)_8_ spacer (S, serine; G, glycine), 47 ± 9% activity was recovered (Fig. 4*C*), corroborating our observation that the TCR tail length is critical for proper positioning (Fig. 3 *F* and *G*). Thus, the TCR signal can be exploited to guide nonnative Fe–S proteins to the CIA machinery for metallocofactor delivery.

## Discussion

N- or C-terminal primary sequence determinants are important signals for subcellular trafficking and posttranslational modifications (35). Herein, we establish that cytosolic and nuclear Fe–S protein maturation is another key process controlled through short linear peptide motifs presented on the solvent-exposed C-terminus. The [LIM]-[DES]-[WF] tripeptide is ubiquitous in the eukaryotic domain of life and consistent with our proposal it serves as a universal signal-directing cytosolic and nuclear Fe–S protein maturation. Since CIA is proposed to biosynthesize [4Fe–4S] proteins (1, 36), we were initially surprised to identify TCR motifs in four bis-histidinyl coordinated [2Fe–2S] proteins, including Apd1 which requires its C-terminal tryptophan for CTC interaction and in vivo function (Figs. 2*B* and 3*A*). Consequently, our data imply that [2Fe–2S] proteins are also matured by the CTC.

Surprisingly, we uncovered “cryptic” TCR motifs in ELP4 and PRIM1 (Fig. 1*E* and *SI Appendix*, Fig. S12), which are nonmetal binding subunits of the elongator and primase complexes. We speculate ELP4 and PRIM1 serve as adaptors for ELP3 and PRIM2, the [4Fe–4S] subunits, similar to Lto1–Yae1 for Rli1 maturation (22). First, PRIM1’s TCR signal is well conserved in Eukaryotes, but absent in archaeal homologs (*SI Appendix*, Fig. S12), following the pattern observed for other TCRs (*SI Appendix*, Fig. S2). Second, PRIM1 was found to be required for formation of the primase–CTC (33), but the molecular basis for this requirement was puzzling. By docking of PRIM1 into the available cryo-EM density of the CTC–PRIM1–PRIM2 complex (33), we found that PRIM1’s TCR signal is properly positioned to interact with the CIAO1 and CIAO2 subunits of the CTC (Fig. 4*D*). This previously unnoted finding not only corroborates our proposal that PRIM1 can function as an adaptor for PRIM2, but it indicates that the TCR motif of clients and adaptors anchors in this region of the CTC (33, 37). Altogether, our data explain how ~30% of clients are identified by the CIA machinery: 19% (6 of the 32 human CIA clients) directly interact with the CTC via their TCR, and an additional 9% (ELP3, PRIM2, and Rli1) depend on an adaptor.

Several diseases (including Friedreich’s Ataxia) are associated with defects in mitochondrial Fe–S protein maturation due to mutations in ISC machinery components. Since disregulation of LTO1 and MMS19 contribute to tumorigenesis (2, 38), we inspected the catalogue of somatic mutations in cancer (39) and identified alterations in 6 human genes (*SI Appendix*, Fig. S13) that would lead to decreased TCR functionality. Like for the ISC machinery, a better understanding of CIA function is critical to decipher how mutations which impact CTC-client interaction could contribute to cancer or deficiencies of medical relevance.

One important question remains unresolved: how do the remaining cytosolic and nuclear Fe–S proteins, those without TCR signals or adaptors, recruit the CTC? Our findings combined with previous data indicate that at least two TCR determinants are present in every CIA client. Here, we have shown that the C-terminal peptide guides clients and adaptors to the CTC, increasing the proximity and positioning of the client with respect to the transient Fe–S cluster presented by the CTC. The successful approach described herein provides a framework for the discovery of additional targeting motifs. Finally, it is notable that just one determinant is sufficient to recruit the CIA machinery, as the [LIM]-[DES]-[WF]-COO^-^ motif can be exploited to engineer cluster delivery to a nonnative Fe–S enzyme (Fig. 4). As deficiencies in cluster maturation frustrate efforts to engineer novel biosynthetic pathways requiring Fe–S enzymes, our study provides a roadmap for overcoming these bottlenecks and demonstrates how an improved understanding of the fundamental biochemistry can impact metalloenzyme bioengineering.

## Materials and Methods

### Bioinformatic Analysis.

Fe–S protein and adaptor sequences were identified by NCBI BlastP searches using yeast and human proteins as query sequences. Alignments were performed on the EMBL-EBI platform, which also provided reference proteomes for C-terminal analysis. Datasets of proteins from OrthoDB were aligned and logos to generate Weblogos.

### ^55^Fe Incorporation into Leu1, Growth Complementation, and Leu1 Enzyme Activity.

Sources, strains, plasmids, culture conditions used in yeast studies, in vivo radiolabeling with ^55^FeCl_3_, and determination of ^55^Fe incorporation into Leu1 were according to established procedures (36). Leu1 expression, purification, and reconstitution were performed as described in *SI Appendix*. Protein concentration was assessed using the Microbiuret method with desoxy- cholate/trichloroacetic acid coprecipitation. Isopropylmalate isomerase activity was measured at 235 nm detecting the formation of 2-isopropylmaleate from 0.2 mM 3-isopropylmalate and normalized using controls. For coupled Leu1 activity assays, NADH formation (340 nm) was monitored in an assay containing 0.2 mM isopropylmaleate, 0.4 mM NAD^+^, 2 mM pyrazol, and 0.4 U isopropylmalate dehydrogenase (*E. coli* LeuB). Mitochondrial succinate dehydrogenase activity was measured from cytochrome *c* reduction at 550 nm (8.9 mM succinate, 1 mg/mL bovine heart cytochrome *c*).

### Affinity Copurification Analysis.

Affinity copurifications were carried out as described (21). For Apd1 copurification strep-tagged Cia1 was incubated with ^SUMO^Met18, Cia2^Δ102^, and Apd1. Truncated Cia2 (Cia2^Δ102^) was used to avoid overlapping on the gel with full-length Apd1 and ^Strep^Cia1. For copurification of Leu1 and SPC-fusions ^SUMO^Met18, ^DT^Cia2 (DT, doubly tagged bait) and ^His^Cia1 were used. Input and Streptactin elution fractions were analyzed by SDS-PAGE and/or Western blot.

Comprehensive procedures for yeast strains, construction of plasmids, culture conditions, activity measurements, radioactive iron incorporation, purification of proteins, affinity copurification, and bioinformatic analysis are compiled in *SI Appendix*.

## Supplementary Material

Appendix 01 (PDF)

Dataset S01 (XLSX)

Dataset S02 (XLSX)

## Data Availability

All study data are included in the article and/or supporting information, plasmids and yeast strains are available upon request.
