# Supplementary material for: Cytosolic iron–sulfur protein assembly system identifies clients by a C-terminal tripeptide
Source: Proc Natl Acad Sci U S A. 2023 Oct 26;120(44):e2311057120. doi: 10.1073/pnas.2311057120 (PMC10623007; doi:10.1073/pnas.2311057120)
Supplement: Supplementary file 1 — Appendix 01 (PDF) [file pnas.2311057120.sapp.pdf]

PNAS

Supporting Information for
Cytosolic iron-sulfur protein assembly system identifies clients by a C-terminal tripeptide

Melissa D. Marquez, Carina Greth, Anastasiya Buzuk, Yaxi Liu, Catharina M. Blinn, Simone Beller, Laura Leiskau, Anthony Hushka, Kassandra Wu, Kübra Nur, Daili J. Netz, Deborah L. Perlstein, Antonio J. Pierik

Deborah L. Perlstein and Antonio J. Pierik
Emails: dperl@bu.edu and pierik@chemie.uni-kl.de

This PDF file includes:

Supporting text
Figures S1 to S13
Tables S1 to S8
Legends for Datasets S1 to S2
SI References

Other supporting materials for this manuscript include the following:

Datasets S1 to S2

Supporting Information Text

Materials and Methods

SUMO peptide carrier (SPC) plasmid construction and purification. The SPC variants were constructed using the pTB146 plasmid (gift from Thomas Bernhardt, Harvard University). DNA encoding the C-terminal tail of Leu1 (FDNVPKRKAVTTTFDKVHQDW; QDW), Nar1 (KDLVSVGSTW), Pol3 (QEKVEQLSKW) and Rev3 (EEALISLNDW) were inserted at the 3'-end of the His-SUMO coding region in pTB146 via Q5 Site-Directed Mutagenesis Kit (NEB) or by gene synthesis (for SPC-Nar1 and SPC-Pol3, GENEWIZ). Plasmids were transformed into an E. coli BL21(DE3). Cells were grown at 37ºC until an OD600 0.7, isopropyl β-D-1-thiogalactopyranoside (IPTG, 0.5 mM) was added, and cells were harvested 4 hours later. For purification, cells (~10 g wet cell paste) were resuspended in 100 mL of Buffer A [50 mM Tris-HCl (pH 8.0), 100 mM NaCl, 5% glycerol, 5 mM ß-mercaptoethanol (ß-ME)] supplemented with 1 mM PMSF (GoldBio), 4 kU DNase nuclease (Fisher Scientific), 5 mM imidazole, and 1 mg/mL lysozyme (GoldBio). Cells were disrupted by sonication and extract clarified by centrifugation. Extract was applied to a 4 mL Ni-NTA resin. The column was washed with >15 column volumes (CV) of Buffer A containing 20 mM imidazole and eluted with Buffer A with 350 mM imidazole. Protein containing fractions were combined, and exchanged into Buffer A via dialysis or gel filtration, concentrated to 10-20 mg/mL via ultrafiltration (Amicon, 10 kDa cutoff filter, Millipore), and stored at -80˚C.

Apd1 cloning and purification. The plasmid with Apd1 cloned into pDONR201 was obtained from the plasmid repository at Arizona State University (DNASU) and inserted via Gateway cloning into the pDEST17 vector, a commercially available gateway destination plasmid for N-terminally His-tagged expression in *E. coli* to create the His-tagged Apd1 (HisApd1) expression plasmid. The W316A substitution was introduced via Q5 Site-Directed Mutagenesis Kit (NEB) (Table S3). All sequences were confirmed by DNA sequencing. For expression, plasmids were transformed into an *E. coli* BL21(DE3) with the ISC operon (a gift from Markus Ribbe,(1)) and cells were grown and induced as previously described (2). For purification, typically 5 g of cell paste was resuspended in 50 mL of Buffer B (50 mM Tris-HCl (pH 9.0), 300 mM NaCl, 5% glycerol, 5 mM ß-ME) supplemented with 1 mM PMSF (GoldBio), one protease inhibitor tablet (Thermo Scientific), 4 kU DNase nuclease (Fisher Scientific), and 5 mM imidazole. Cells were lysed by addition of CelLytic Express (2.5 g, Sigma Aldrich) and extract was clarified by centrifugation. After batch absorption for 1h, the Ni-IMAC resin (GoldBio) was collected, washed with 20 CV of Buffer B with 20 mM imidazole, and eluted in Buffer B with 250 mM imidazole. Protein containing fractions were combined, exchanged into storage buffer [50 mM Tris-HCl (pH 9.0), 100 mM NaCl, 5% glycerol, 5 mM ß-ME], concentrated to 4-7 mg/mL via ultrafiltration (Amicon, 10 kDa cutoff filter, Millipore), and stored at -80°C.

Affinity co-purification analysis. Leu1 variants in Fig. 3, C and F were created in previously described Leu1 plasmid encoding a TEV-cleavable N-terminal His-tag (^{HisTEV}Leu1) (3). ^{HisTEV}Leu1 variants were created by Q5 mutagenesis (NEB, Table S3) and confirmed by DNA sequencing. IMAC purification of ^{HisTEV}Leu1 was carried out as described (3). In the co-purification assay, Met18 with a N-terminal His-SUMO tag (^{SUMO}Met18); singly tagged Cia1 with an N-terminal His-tag (^{His}Cia1) or double tagged Cia1 (^{DT}Cia1) with an N-terminal His-TEV-Streptag; doubly tagged Cia2 (^{DT}Cia2) with a thrombin-cleavable N-terminal Strep-tag and a HRV3C-cleavable C-terminal His-tag; and truncated Cia2 (Cia2$^{\Delta102}$) in which the first 102 aa in Cia2's N-terminus and a C-terminal His-tag, were purified as previously described (3, 4). When required, the His-tag was removed from ^{DT}Cia1 via treatment with TEV protease and isolation of resulting singly Strep-tagged Cia1 (^{Strep}Cia1).

Affinity copurification assays were carried out as previously described (3). In all cases, data presented is representative of a minimum of three independent experiments. Briefly, a Strep-tagged bait protein, ^{DT}Cia2 (for Leu1 and SPC-fusions) or ^{Strep}Cia1 (for Apd1), was mixed with an equimolar amount of each CTC subunits (^{SUMO}Met18, ^{His}Cia1 or Cia2$^{\Delta102}$, as appropriate) and a TCR-tail prey protein (Leu1, Apd1 or SPC-fusions). For Apd1 experiments, the truncated Cia2 (Cia2$^{\Delta102}$) was used due to the close molecular weights of the full length Apd1, ^{Strep}Cia1, and Cia2. For SPC

fusions, roughly 3-fold molar excess over bait protein concentrations were used. In all experiments, a “no-bait” negative control was completed in parallel to detect any nonspecific binding to the resin. The samples were incubated for 1 h 4˚C and then chromatographed through streptactin resin. Input and Elution fractions were analyzed SDS-PAGE (12% for experiments with Leu1, 15% for SPC-fusions, 20% for Apd1). For Western blot analysis, proteins were transferred using a Trans-Blot Turbo Mini PVDF Transfer pack and Transfer System with the mixed molecular weight protocol in the manufacturer’s instructions (Bio-Rad). The membrane was blocked with 5% casein in TBST and probed with Anti-His antibody (Cell Signaling Technology). Bands were detected via chemiluminescence, according to manufacturer's instructions.

Yeast Strains and Cell Growth. The *Saccharomyces cerevisiae* strain W303-1A (MATa, *ura3-1*, *ade2-1*, *trp1-1*, *his3-11,15*, *leu2-3,112*) was used as the wild-type strain. Yeast strains are summarized in Table S4. To convert the W303-1A strain into a *LEU2*$^+$/Δ*leu1* strain, first homologous recombination with a *leu2* PCR fragment followed by selection on SC glucose medium lacking leucine was carried out. The *leu2* PCR product was obtained by fusion PCR of *leu2* fragments A, B and C (see Table S5). Yeast strains, Δ*leu1*, *LEU2*$^+$/Δ*leu1*, Gal-NFS1/Δ*leu1*, Gal-*NAR1*/Δ*leu1*, Gal-CIA1/Δ*leu1*, and Gal-CFD1/Δ*leu1*, were constructed by homologous recombination in which the coding region of the *leu1* gene was replaced by a natNT2 cassette amplified from pYM17, omitting the 6xHA tag (5). Gal-*POL3* was constructed by homologous recombination in which the nucleotide-upstream promoter region was replaced by the natNT2 cassette from pYM-N27 (5), including a GAL promoter sequence. The correctness of the insertion was assured by PCR analysis of genomic DNA. Cells were grown on rich (YP) or minimal (SC) media containing 2 % (w/v) galactose or glucose as carbon source with the appropriate markers.

Yeast expression plasmid construction. For *POL3* cloning, the *E. coli* strain NEB5 F’I^q was used. All other genes were cloned using the *E. coli* strains NEB5 or NEB10. *E. coli* genomic DNA was used as the template to amplify *leuC* and *leuD*. For the other genes (*POL3, NAR1, LEU1, APD1*), *S. cerevisiae* genomic DNA was the template for PCR. Information, including primers (Table S6) and plasmids (Table S7), are provided and when not specified otherwise, standard cloning procedures were used. Variants were generated by site-directed mutagenesis with primer design according to Zheng *et al.* (6). For C-terminal truncations, the appropriate codon was replaced by a stop codon. Successful construction of all plasmids was verified by Sanger sequencing.

The *POL3* coding region and the 500 bp at the 5’ end of the start codon was inserted into the SacI and EcoRI sites of pRS416-MET25$_{prom}$-MCS-CYC1$_{term}$ (maintaining the *CYC1* terminator) (7). During cloning and mutagenesis of *POL3*, growth for plasmid minipreps required incubation of agar plates at room temperature for 2 days until colonies appeared and cultivation in liquid media at 30 °C.

For *APD1*, its coding region, the 526 bp natural promoter, and 327 bp terminator regions were cloned into the SacI and KpnI sites of pRS416-MET25$_{prom}$-MCS-CYC1$_{term}$.
The *NAR1* coding region, along with its natural promoter (591 bp) and terminator (558 bp) were cloned into SacI and KpnI sites of pRS416-MET25$_{prom}$-MCS-CYC1$_{term}$. For the Nar1-encoding plasmid under control of the *MET25* promoter, the *NAR1* coding region and its terminator (558 bp) were cloned into SpeI and KpnI sites of pRS416-MET25 (7). For the Nar1 plasmid with *NBP35* promoter, the promoter region of the *NBP35* gene (508 bp) was inserted between the SacI and SpeI sites of pRS416-MET25$_{prom}$-Nar1-NAR1$_{term}$.

The *LEU1* coding region, along with its natural promoter (552 bp) and terminator (512 bp) regions, were cloned as two PCR fragments employing the natural SalI site (883-888) in the *LEU1* coding region. First, the SalI-NgoMIV fragment was cloned into pRS416-MET25$_{prom}$-MCS-CYC1$_{term}$, followed by the SacI-SalI fragment. Variants with other promoter regions were generated by introducing a SpeI site just before the ATG start codon of *LEU1*, followed by cloning PCR fragments of the promoter regions of the *MET25* (412 bp), *TDH3* (649 bp), *RET2* (500 bp) and *RPL18B* (500 bp) genes between SacI and SpeI sites. For pRS416-TDH3$_{prom}$-Leu1-TDH3$_{term}$, an XhoI site was introduced immediately following the *LEU1* stop codon and a BamHI site was inserted after the

LEU1 terminator. Then, a PCR fragment of 449 bp corresponding to the *TDH3* terminator was cloned into the XhoI and BamHI sites.

For generation of pRS416-TDH3$_{prom}$-Leu1-TDH3$_{term}$ with the C-terminal 19 amino acids of the yeast Leu1 replaced by the C-terminal 19 amino acids of the homologs of *S. pombe* or *A. nidulans,* a BglII was introduced by mutagenesis at nucleotides 1847-1852 of the *leu1* gene, creating a silent mutation. Then, synthetic genes corresponding to the BglII sequence plus nucleotides 1853 to 2280 of yeast *LEU1*, and yeast codon optimized nucleotides encoding the 19 C-terminal amino acids of the homologs plus a stop codon and the XhoI sequence were cloned into BglII/XhoI sites of pRS416-TDH3$_{prom}$-Leu1-TDH3$_{term}$.

For construction of the yeast expression vector for LeuCD (pRS426-FBA1$_{prom}$-LeuC-linker-LeuD-Leu1-CT-FBA1$_{term}$), *E. coli leuC* and *leuD* genes were each amplified and separately cloned into the SpeI/XhoI sites of the MCS of pRS424 and pRS426, respectively. To create the backbone of the shuttle vector, the *TDH3* promoter of vector pRS426-TDH3$_{prom}$-MCS-CYC$_{term}$ (7) was replaced by 1000 bp of the *S. cerevisiae FBA1* promoter, using SacI and SpeI sites. Then, the *CYC1* terminator was exchanged for the *FBA1* terminator (1000 bp), using XhoI and KpnI sites. To create pRS426-FBA$_{prom}$-LeuD-Leu1-TCR, the DNA fragment encoding the yeast Leu1 C-terminus (last 30 amino acids, Leu1-TCR) was cloned into EcoRI/BamHI of the multiple cloning site of pRS426-FBA1$_{prom}$-MCS-FBA1$_{term}$ followed by cloning of *leuD* from *E. coli* (using pRS426 with *E. coli leuD* as template) into pRS426-FBA1$_{prom}$-Leu1-TCR-FBA1$_{term}$. To introduce *leuC* and the linker region of Leu1 (amino acids 481-543) concomitantly, PCR overlap extension was used (8). In the first PCR reaction (I), *leuC* (in pRS424) was amplified such that the forward primers introduced a SpeI restriction site and the reverse primer introduced 15 nucleotides corresponding to the 5' end of the *LEU1* linker. In the second PCR reaction (II), the *LEU1* linker was amplified (from pRS416-*TDH3*$_{prom}$-Leu1-*TDH3*$_{term}$). The forward primer introduced 20 nucleotides corresponding to the 3'end of *leuC* and the reverse primer appended an SpeI restriction site. An equimolar amount of PCR-product, I and II, were mixed and hybridized by using a PCR protocol without dNTPs. In a final PCR reaction, the primers flanking the SpeI sites were used to amplify the product, which was subsequently cloned into the SpeI restriction site of pRS426-FBA$_{prom}$-LeuD-Leu1-TCR, generating pRS426-FBA$_{prom}$-LeuC-LeuD-Leu1-TCR-FBA$_{term}$. To test the effect of the Leu1-TCR, a stop codon was introduced after the *leuD* coding region by site-directed mutagenesis. To create the QDW tail, primers were used to insert the tripeptide coding region plus a stop codon immediately following *leuD*.
To shorten the TCR tail from 29 to 10 amino acids (770-779 amino acids of Leu1), Q5 mutagenesis was used to delete the region corresponding to amino acids 750-769 of Leu1-TCR and install an Xho1 site. The resulting PCR product was digested with XhoI cut and ligated. For the construct with the SSG linker sequence substituting the Leu1-TCR, a synthetic DNA sequence encoding for the eight SSG repeats and the codons for QDW was chemically synthesized and cloned into the XhoI/EcoRI sites of 426-FBA1$_{prom}$-LeuC-linker-LeuD-Leu1-TCR-FBA1$_{term}$.

Protein expression and purification of Leu1 variants for activity measurements. Yeast Leu1 was cloned into pET28a (Novagen), fused to a N-terminal hexa-histidine tag for heterologous expression and purification. After transformation into BL21 (DE3) cells (NEB), an overnight pre-culture was diluted (2% inoculum) into 2 liters of LB medium containing ampicillin and 3% (v/v) ethanol p. a and allowed to grown at 37 °C and 200 rpm/min shaking. When the culture reached an OD_{600} of 0.5, the induction was started by addition of IPTG (0.5 mM), followed by incubation at overnight at 30 °C. Cells were opened with French Press and purified using Ni-IDA (Cube) according to the manufacturer's instructions. The protein eluates were desalted using a Sephadex G-25 column (GE Healthcare) equilibrated with 25 mM Tris-HCl, pH 8.0, 300 mM NaCl. The purified proteins were shock-frozen and stored at -80 °C until use.

Reconstitution of recombinant purified apo-Leu1 was performed in an anaerobic chamber (Coy Laboratory). Approximately 50 µM of Leu1 was reduced with DTT (15 mM, end concentration) for 45 min. After addition of 5 molar-equivalents of ammonium iron (III) citrate and development of a red color the same equivalents of lithium sulfide were slowly pipetted into the reaction tube. The

sample was incubated for 2-4 h and the Leu1 activity was tested during this time without desalting the sample.

^{55}Fe incorporation into Leu1. Leu1 or mutants thereof cloned into pRS416 plasmids under the control of the endogenous *LEU1* promoter were transformed into yeast cells, as indicated in figure captions. *In vivo* radiolabeling with $^{55}FeCl_3$ and determination of ^{55}Fe incorporation into Leu1 beads was carried out as described (9). Briefly, freshly transformed cells were grown on iron-poor SC medium for 40 h supplemented with the appropriated carbon source. After incubation with $^{55}FeCl_3$ and 1 mM ascorbate for 2 h in SC iron-poor medium, cell extracts were prepared by using glass beads. The Leu1 protein was immunoprecipitated by using polyclonal antibodies against Leu1 bound to Protein A-Sepharose beads. The amount of protein-associated radioactivity was assessed via liquid scintillation counting.

Growth complementation. Growth complementation assays used the W303, Gal-*POL3*, Δ*apd1*, Gal-*NAR1* and *LEU2*$^{+}$/Δ*leu1* strains transformed with appropriate plasmids (see figure legends). Transformants were grown in liquid SC medium supplemented with galactose or glucose (2 %) at 30 °C for 40 h (Δ*apd1*, *LEU2*$^{+}$/Δ*leu1* and Gal-*NAR1*, including control W303), or for 16 h (series Gal-*POL3*, including control W303). At this point, the cultures were diluted in SC medium to an OD_{600} of 0.5 and 5 µL aliquots were submitted to a 10-fold serial dilution and spotted into SC agar plates supplemented with glucose or galactose carbon source. SC agar plates with methyl methane sulfonate or gallobenzophenone at the indicated concentration were freshly prepared. The plates were incubated for 48 h at 30 °C and photographed. At least three experiments using independently transformed yeast cells were performed.

Enzyme activity determination. Protein determination for activity in crude extracts was performed using the Microbiuret method with desoxycholate/trichloroacetic acid coprecipitation (10, 11). Isopropylmalate isomerase and succinate dehydrogenase activity were determined in freshly prepared yeast or *E. coli* cell extracts. Yeast cell lysates were prepared using the glass bead method and clarified by centrifugation, whereas *E. coli* cell lysates were prepared using a French Press. For Leu1 activity, the reaction was conducted in a buffer containing 20 mM Tris-HCl, pH 7.4 and 50 mM NaCl. The formation of isopropylmaleate after addition of 0.2 mM 3-isopropylmalate was followed by the increase of absorption at 235 nm. To correct for variations in growth conditions and deviations from the ambient temperature (22 °C), a wild type Leu1 or LeuC-linker-LeuD-TCR (variant 12, Fig. 4C) control was measured in parallel and used to normalize the series measured on different days.

For the coupled assay of Leu1 activity, *E. coli* isopropylmalate dehydrogenase *(leuB*) was cloned into the BamHI and HindIII sites of MCS1 of pET-Duet1 and purified by Ni-NTA using standard procedures. Assays contained 20 mM Tris-HCl (pH 8), 50 mM KCl, 10 mM $MgCl_2$, 1 mM DTT, 0.2 mM isopropylmaleate, 0.4 mM NAD^+, 2 mM pyrazol and 0.4 U LeuB. The absorbance increase from NADH formation at 340 nm was followed.

The succinate dehydrogenase activity in isolated mitochondria was determined by measuring the reduction of cytochrome *c* at 550 nm in a reaction containing 8.9 mM succinate and 1 mg/mL bovine heart cytochrome *c* in a buffer containing 50 mM Tris, pH 8.0 and 50 mM NaCl. The background activity was assayed by measuring the increase of absorbance in the same reaction containing 14.5 mM malonate and 1 mM potassium cyanide.

Bioinformatic analysis. Fe-S proteins and adaptor amino acid sequences were collected by NCBI BlastP searches using yeast und human sequences as query. Amino acid sequence alignment was carried out with ClustalO on the EMBL-EBI server (12). The same server was used for the download of reference proteomes for the analysis of C-termini (Data S1). For analysis of the C-termini of *Homo sapiens* (13), *Saccharomyces cerevisiae* (14), *E. coli* (15), *Methanocaldococcus jannaschii* (16), and *Arabidopsis thaliana* (17) the published Fe-S protein inventories were manually updated. Datasets for the generation WebLogos (18) were downloaded by selection of the appropriate taxonomic class of organisms in the OrthoDB catalogue of orthologs (19). Sequence fragments

lacking N-terminal or C-terminal regions and excessively long sequences due to erroneous translation and/or wrong assignment of introns were manually removed. Then the unaligned C-terminal 20 amino acids were submitted to the Weblogo server for data in Fig. 1E, and fig. S3. The Apd1 collection was from the phylogenetic tree as described in Stegmaier et al. (2).

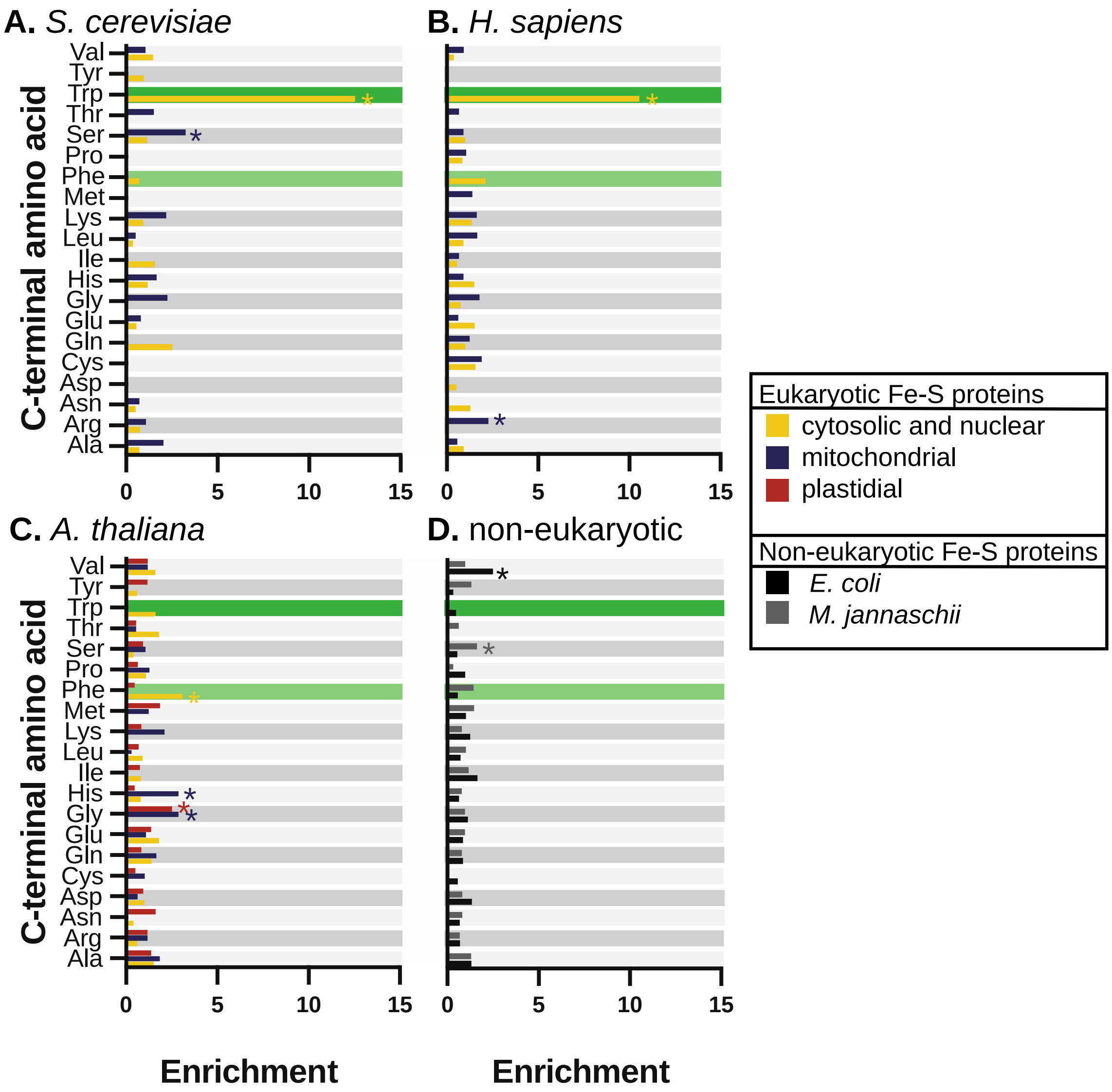

Fig. S1. Bar graphs comparing enrichment in C-terminal amino acids in the Fe-S proteomes of yeast (**A**), humans (**B**), plants (**C**), and non-eukaryotic organisms (**D**). For Panels (A-C), the CIA proteome is in yellow, the mitochondrial Fe-S proteome is purple, and plastidial Fe-S proteome is red. In Panel D, the *Methanocaldococcus jannaschii* Fe-S proteome is in light gray and *Escherichia coli* Fe-S proteome is black. In each dataset, the C-terminal amino acid with the highest enrichment is marked with an asterisk. A terminating W (dark green) or F (light green) is exclusively enriched in the CIA proteome.

A

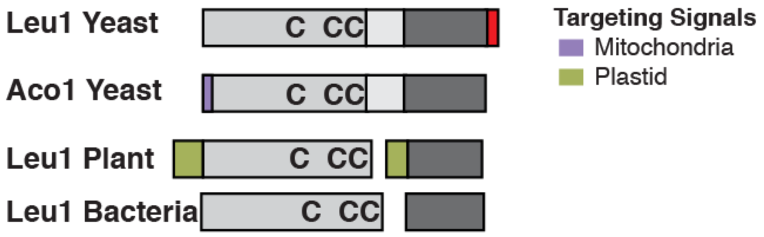

B

```
Saccer_Leu1      ANKGGKLCVDLPNQKILDSDGNVLVDHFEIEPFRKHCLVNGLDDIGITLQKEEY 735
Aspnid_Leu1      ARAGREVEVDLVNQEIKDEAGNKL-ASFDVDAFRKHCLINGLDDIGLTLQMEDK 720
Neucra_Leu1      AAAGREIEIDLPNQLIKDADGNTL-CEFEVEEFRKHCLVNGLDDIGLTMQMEDK 727
Schpom_Leu2      AENQVKFSVDLVNQTITYGDKQ---VKFDVEPFRKHCLVNGLDDIGLTLQKETM 719
Esccol_LeuD      ANPGIHFDVDLEAQEVKA-GEKT--YRFTIDAFRRHCMMNGLDSIGLTLQHDDA 188
Pseput_LeuD      ANPGYQLTIDLQAQAVTRPDGKV--LHFEIDAFRKHCLLNGLDDIGLTLQDSDA 195
Strmut_LeuD      LKPTDEVTVNLFEQKIYSPVGD---FSFDIDGEWKHKLLNGLDDIGITLQYEDL 183
Aratha_LeuD3     CTTGDVATVELRE-------GDSILINHTT---GKEYKLKPIGDAGPVIDAGG- 235
                        ::*          .    .      :. :: :.. * .::

                                           ~30-40 aa
Saccer_Leu1      ISRYEALRREKYSFLEGGSKLLKF----------DNVPKRKAVTTTFDKVHQDW 779
Aspnid_Leu1      IAKFEAKRTLDTPWLDGKAYLKRGRTGGSNMVKAAPVPKTN--RGDVKGEPLEW 772
Neucra_Leu1      IAEYEAKMSQQTPWLDGRAYLKRKGQGGKLVAKAVPVPKTN--RGEEKKEPLEW 779
Schpom_Leu2      IDAFEAAREENFPWMNIKRSRARL----------SPVKSNK-----QSSSRNDW 758
Esccol_LeuD      IAAYEAKQPAFMN----------------------------------------- 201
Pseput_LeuD      IKAFEAKHRASQPWLFRDA----------------------------------- 214
Strmut_LeuD      IAQYEQNRPSYWH----------------------------------------- 196
Aratha_LeuD3     IFAYARKA------------------G-------MIPSAAA------------ 251
```

C

```
Saccer_Nar1      CPGACMNGGGLLNGEQNS--LKRKQLVQTL-NKRHGEELAMVDPLTLGPKLE    460
Schpom_Nar1      CPGGCINGGGQLPFPSVERIVSARDWMQQV-EKLYYEPGT-------RSV-D    495
Aratha_NARFL     CPAGCLNGGGQIKPKTG---QSQKELIHSL-EATYMNDTT----LNTDPY-Q    432
Dromel_NARFL     CPSGCINGGAQIRPTTG---QHVRELTRKL-EELYQNLPR------SEPE-N    435
Danrer_IOP1      CPSGCLNGGGQLKPSAD---QSNKELLQQV-EEVYRSEHP------SVPE-D    435
Homsap_NARFL     CPSGCLNGGGQLQAP-D---RPSRELLQHV-ERLYGMVRA------EAPE-D    434
Xenlae_IOP1      CPSGCLNGGGQIKAE-G---EVGKDLLQRV-EELYNSMRS------ERPE-E    434
Chlrei_HydA1     CPAGCVGGGGQPRSTDKA--I-----TQKRQAALYNLD----EKSTLRRSHE    457
Clopas_Hyd       CHGGCVNGGGQPHVNPKD--LEKVDIKKVRASVLYNQD----EHLSKRKSHE    544
Desvul_Hyd       CPGGCVCGGGQPVMP-GV--LEAMDRTTTR---LYAGLK---KRLAMASA--    413
                 * ..*: **.                       :

                                                         5-6 aa
Saccer_Nar1      EAAARPLS---------------LEYVFAPVKQAVEKDLVSVGSTW    491
Schpom_Nar1      QSAVSYMLEQWVKDPT--LTPKFLHTSYRAVQTDNDN-PLLLANKW    538
Aratha_NARFL     NPTAKRLFEEWLKEPGSNEAKKYLHTQYHPVVKSVT--S--QLNNW    474
Dromel_NARFL     -SLTKHIY NDFLDGFQSDKSYDVLHTRYHDVVSELSI-S-LNINW    477
Danrer_IOP1      DSQVAELYQSWLESVGEEKARQLLHTQYHAVE-KTAN-G--LSIKW    477
Homsap_NARFL     APGVQELYTHWLQGTDSECAGRLLHTQYHAVE-KAST-G--LGIRW    476
Xenlae_IOP1      WKQVTQIYRDWLQGCDSPRARQVLHTQYHEVE-KINS-G--LTIKW    476
Chlrei_HydA1     NPSIRELYDTYLGEPLGHKAHELLHTHYVAGGVEEKDEKK------    497
Clopas_Hyd       NTALVKMYQNYFGKPGEGRAHEILHFKYKK----------------    574
Desvul_Hyd       NKA-------------------------------------------    421
```

D

```
Homsap_RSAD2     LERHKEVS-----CLVPESNQKMKDSYLILDEYMRFLNCRKGRKDPSKSILDVG  328
Ornana_RSAD2     LERHKEIA-----CLVPESNQKMKDSYLILDEYMRFLNCRNGRKEPSRSILDVG  332
Trivir_RSAD2     CERHSSQT-----CLVPEPNRLMAKSYLILDEYMRFLDRNG--QQPSKSILEVG  270
Nemvec_RSAD2     ISRHASLE-----CLVPESNEKMQNSYLILDEYMRFLDCTGGSKSPSKSILDVG  260
Strapo_Hom       VRRHSWVA-RYGIDVVPENNDAMTGSYAMLDPAGRFFDNVRGSYTYSASILRCG  267
Lepkan_Hom       LKNHKELA-KFGLKIVPENNSAMTNSYLMVNPDGCFYYNHNGKYLLSDPILDVG  259
Thesic_MoaA      IERHEDL------NPIAEDNNLMTESYLMMDPYGRFYDEESQLENIRPSLLDAP  263
Methol_MoaA      IKTNKDIVLSSGDSPVFETNDDMVDSYLMIGPNGSVIKNSNMEHAVEDMRIN-G  252
                    :           : * *   *   ** ::.     .                :   .
```

```
                                                6-7 aa
Homsap_RSAD2     VEEAIKFSGFDEKMFLKRGGKYIWSKADL-----KLDW    361
Ornana_RSAD2     VQEAIKFSGFDEKMFLKRGGKYVWSKAEL-----NLQW    365
Trivir_RSAD2     VQQALQAVFWDEEAFVERGGIYDWNKSSCSSDSKDLEW    308
Nemvec_RSAD2     VDQAMKFSGFDEKMFLKRGGKYVWSKADM-----KLDW    293
Strapo_Hom       VPAALRQITLDRSKYLARGGQYDWSAPLPVLARGATD-    304
Lepkan_Hom       IETALKQIHFSKDKFKKREGFYII--------------    283
Thesic_MoaA      FEEAISGVKFDFSKFVLRGGIYNWRRAEDEV-------    294
Methol_MoaA      IEEI--DSIVDWKKYHQRGGD-HWISQ-----------    278
                           . . :  * *
```

E

```
Saccer_Ncs6      KNLEAVRPSCIIDIIQSGENLALKAKKSNAGKRVVKFVDGNRCARCGYLSSNNI   319
Homsap_Ctu1      KRLEAARPSAVLDLVHSAERLALAPA--------ARPPRPGACSRCGALASRAL   298
Danrer_Ctu1      KDLESVRPSAIIDVIHSGETLSVKEG--------VKMPVQGTCSRCGYISSQAL   295
Aratha_Ctu1      KDLERIRPRAILDIIKSGEDFRIATT--------TKMPEQGTCERCGYISSQKW   322
Pyrhor_TtuA      NELEEKRPGTKFNFVRGYLKKKKLFE---PE---IKEKEIKECKICRMPSSGDI   283
Metmar_NcsA      DNLEKNHPGSKYSIVRGYERLLEHIE--LPGY-------TGECKICGDLSATEV   293
Halvol_NcsA      LKLEENHPGTRHSIMAGYEELAELTA---REYRGEGRVDLNDCERCGSKTAGDV   308
Themar_TtuA      RELEEEQPGITLNFYLGFLKRKK--E---PK---FEVEGLRECKECGYPTTAEV   285
                   **  :*     ..  .                          *  *   ::
```

```
                                     30-40 aa
Saccer_Ncs6      CKACMLLEGLEKSRAQVAIENDTSADGAALKLRAL-------------EKLSF   359
Homsap_Ctu1      CQACALLDGLNRGRPRLAIGKGRRGLDEEAT-PGTPGDPARPPASKAVPT--F   348
Danrer_Ctu1      CKSCVLLEGLNRGLPKLGIGKHHRLHGKILAQEPLTEQ-----EERKLKAVDF   343
Aratha_Ctu1      CKACVLLEGLNRGLPKMGIGRPRGVNGDHNKETKKPGSVAKSIESKQCGSLDF   355
Pyrhor_TtuA      CAFCKFWGLKKEINFKVSS---------------TDEEPFGP-----------   310
Metmar_NcsA      CKVCSYLKNLGILEKSK-FEKI-------------------------------   314
Halvol_NcsA      CRKCRLIESIEAV----------------------------------------   321
Themar_TtuA      CSFCRLRKQVEKRKNKTPA----------------------------------   304
                 *  *
```

F

```
Neucra_Nit6        FDVEERDDGMVYIKLPPVDELDRELGTKKWMVKKGEAGEGQLR--ELDELNKSK   1134
Aspnid_NiiA        FPVEEREDGWIYMKLPPVEELDSVLGTEKWKVKKGEAV---DPFEAYDKK--YS   1077
Magory_NiiA        FEVEERPDGHVYAKLPPVEELDAALGTSKWKVKLGDAGEGEHPFAELDRKIAFK   1116
Talsti_NiiA        FPAEEREDGWVYLKLPSTEELDSVLGTEKWKIKSTESE---DPFEKVDKK--YK   1066
Blamar_Nir1        FPVKIE-ADGVYLELPPETVLDAELATDKHCIRGCDAVKALQLA--------EA   1000
Nitbac_Nir1        FPVRVE-GEDVYLNLPESDVLNPLLATEIGCTLATS----------------CT   976
Brevol_Nir1        FKVKVE-ESQVFVQLPPQETLNAKLATKLHCISACDADKSRQMS--------CV   1001
Gimpan_Nir1        FNVKVDEQENVYLELPEAEVLDELINQTV-----------------------CA   984
                   *  ..      :: :**    *:  :

                             40-45 aa
Neucra_Nit6        GVEGKKGRRGRKPGASEAGKEVGKKLVEAVG-GGGCGGPGLEW 1176
Aspnid_NiiA        GMKGKRA------GA---------KGIEGSK-PTRSPSNTIDW 1104
Magory_NiiA        GLRAKK--VGLRPTAAT--LPLKSKNLAVAV-GGGCGS-APDW 1153
Talsti_NiiA        GTRGKKI------GD---------VHVAVKEVSPCGGGNSIDW 1094
Blamar_Nir1        ------------------------------------------- 1000
Nitbac_Nir1        G------------------------------------------ 977
Brevol_Nir1        GS----------------------------------------- 1003
Gimpan_Nir1        GAH---------------------------------------- 987
```

G

```
Saccer_Apd1      YLKKVFDSKLQEHGLYRDNSDYRAEGVKIAFVNHVGGHKFAANVQIYLRN----        271
Yarlip_Apd1      ILKKEFDAQLRDKQ---------IHDVEVAFVSHVGGHKFAANALIYLS-----        279
Picpas_Apd1      IIKKEMCIHLRDHDLYRDLGDDRPGGVQVHFINHVGGHKYAANVLIYLK-----        260
Candub_Apd1      IMKREMDNYLEELDLKRNFGDNRPNGIQTEFINHIGGHKYAANVIIYLKK----        267
Saccer_Aim32     DIIAAFQD----EKL--------FPENNLALISHIGGHIFAGNVIFYKLFGREK        269
Yarlip_Aim32     LILGEMDEIKQEYA--RDTSRDSPRDIHTALISHIGGHAFAGNVLLFSGQ----        261
Picpas_Aim32     LLKKEFELVLNKEGL-LYNKYKNPGGIKVGIISHVGGHAFAGNVIYFNTA----        243
Candub_Aim32     QLESEFNQVLARHNL--------QGTIYTGQISHVGGHAYAGNVLYYPKD----        259
                  :   :                        :.*:***  :*.*.   :

                                                                4-6 aa
Saccer_Apd1      ---PNTLIWLGRVTPTIVPSIVEHLIVPEEPTLPFPEKVRCIKKYQ---SW  316
Yarlip_Apd1      ---TGESIWLARVGPEHVCAIIDEVIEKG-K--VFPELVRSVAKCQ--LDW  322
Picpas_Apd1      ---SGANIWLARCNPFNVKPIIEETILGGGK--VWPEHVRLVQKSTKPLQW  306
Candub_Apd1      ---SGKNIWLGLCKPNNIKPIVDECILGDGR--IWPDKVRLIQKF-DPIEW  312
Saccer_Aim32     MQNKLDSLWFGKVYPHNLKLLCENL-ENGKI---IDEMYRGGISMN-----  311
Yarlip_Aim32     ---TGSSSWFGRVRPEHIQGLVKEW-NDGRI---VKELYRGSFAD------  299
Picpas_Aim32     ----GQSIWYGRVFPDKVQGIVNQTVENKTI---IQELYRGQI--------  279
Candub_Aim32     CQTSKDFIWYGRVFPKDVQGIVESTIVNKEI---IKDLFRGDIE-------  300
                         * .    *  :  : .              :  *
```

H

```
Dromel_Nev     VHEFYGPRWIAP----LMKIFI-YGESLMFERDIKIWNHKVFNRNPILAKEDAS     402
Xenlae_Nev     SHSIYYQKNIPA----LIPKFILKAECIQFERDVMIWNNKKYISKPMLVKEDAA     426
Danrer_Nev     SHTIFYQSSIPP----LVPKFILRAECIQFERDVMIWNNKTYISKPMLVKEDSA     432
Bommor_Nev     IHRVYSPAYNAP----V-GAFLVRCEAYMFERDVTIWNSKRFVSAPAYVKTDKT     424
Nosspe_KshA    HLIISFKKSGSGFDNSDLEDMILQAIAIDAEKDLPILEHKVYQSSPVLCEDDGP     309
Mycspe_KshA    RYTFFVLTEPETGEMTRMGMGFVRDFCKQIEQDIPIWENKVFRDRPQLARGESA     308
Halmar_KshA    RWTLLVRKEIED----LAGDDVMKGIIDGLSDDYPIWANKVHRRRPVFCREDKP     302
Sorcel_KshA    RVAFWLPSD----GSQRLGQALAAEICRQIEEDIPIWENKIYRPQPVLSAGEKG     295
                  .                 .        . *  *   * .   *     :

                                          3-9aa
Dromel_Nev     IKKFRLWFSQFYSSNSKIYS--EATNIGW   429
Xenlae_Nev     IQKHRRWFSQFYSNNSPQITF-QQEGLDW   454
Danrer_Nev     IQKHRRWFSQFYSENSPRLRY-QHDTLDF   460
Bommor_Nev     IRTFRNWFGQFYSEHSLGFRDALQNPLDW   453
Nosspe_KshA    IMQYRRWASQFYSPVPSNSKIPQYQS---   335
Mycspe_KshA    ITEFRAWADQSYEQNSSKESTLASQ----   333
Halmar_KshA    LVTFRKWVRQFYVTSQDQQEIA-------   324
Sorcel_KshA    IMTLRKWSSQFL-----------------   307
               :   * *  *
```

I

```
Schsti_Cmo1    DFNLCQLTQKNLMEGIYSSGYLHPTKERGVLYYQGVVRDMVKKHFALEAAAGKP     411
Pickud_Cmo1    DYNLCEATQKNLNNGIYKTGYLHPTRERGVLHYQSLVRDLVKKHYAIEEAAGHP     410
Canten_Cmo1    DFNLCQLTQKNLNEGVYSSGYLHPLKERGVLFYQNLVRDMVKEHFALEQAAGKP     414
Clalus_Cmo1    DFNLCQLTQKNLNQGIYGSGFLHPTKERGVLHYQGLVRDLVKEHLALEEKRGKI     407
Spiole_CMO     DVVLCESVQRGLETPAYRSGRYVMPIEKGIHHFHCWLQQTLK------------     439
Glymax_Cmo     DIVLCEGVQKGLQSPAYRVGRYAPTVEQAMHHFHCLLYENLAK-----------     418
Silpal_Cmo1    DILICEQVQKNLEVGIYQEGILHPERENGVEYFHSLVRKAITNLKGNRDVKNSI     353
Rueare_Cmo1    DIRLVESVHRGLKSRGYRPGPLVLDPACGVMSEHSIARLQQWMREAVDTDSSG-     366
               *  : :  .::.*     *  *         .:   :

                                40-45 aa
Schsti_Cmo1    KPINPAFVGRNQKNEEIEELEAICNSLECSSGNSSKEFEW     451
Pickud_Cmo1    HPVNPAALSASQ-SKDAEELEKMCAGIECMGSTPAEGLEW     449
Canten_Cmo1    KPINPAIISNKKANKDVQELEEICNQLECGSSSGSSELQW     454
Clalus_Cmo1    KIVNPAFLGEAALNDSVKELNELCNKLECTSSEKTAGIDW     447
Spiole_CMO     ----------------------------------------     439
Glymax_Cmo     ----------------------------------------     418
Silpal_Cmo1    SI--------------------------------------     355
Rueare_Cmo1    G------------VDDGRHL--------------------     374
```

Fig. S2. A terminating in W/F is conserved in CIA substrates (bold) at the end of a C-terminal extension (boxed with length of extension noted), but not in eukaryotic homologs with organellar targeting sequences, nor in Bacterial or Archaeal homologs. (**A**) Cartoon of isopropylmalate isomerase and aconitase in Fungi, and isopropylmalate isomerase subunits in Plants and Bacteria. (**B**). Amino acid sequence alignment of C-termini of fungal Leu1 sequences (Saccer, *Saccharomyces cerevisiae*; Aspnid, *Aspergillus nidulans*; Neucra, *Neurospora crassa*; Schpom, *Schizosacharomyces pombe*, historically called Leu2) compared with bacterial LeuD (Esccol, *Escherichia coli*; Pseput, *Pseudomonas putida*; Strmut, *Streptococcus mutans*, PDB 2HCU) and plastidial LeuD3 (Aratha, *Arabidopsis thaliana*). (**C**) Amino acid sequence alignment of C-termini of eukaryotic Nar1/IOP1/NARFL sequences (Saccer, *Saccharomyces cerevisiae*; Schpom, *Schizosaccharomyces pombe*; Aratha, *Arabidopsis thaliana*; Dromel, *Drosophila melanogaster*; Danrer, *Danio rerio*; Homsap, *Homo sapiens*; Xenlae, *Xenopus laevis*) and Fe-Fe hydrogenases (Chlrei, *Chlamydomonas reinhardtii*; Clopas, *Clostridium pasteurianum*; Desvul, *Desulfovibrio vulgaris*). (**D**) Amino acid sequence alignment of C-termini of eukaryotic Viperins (Homsap, *Homo sapiens*; Ornana, *Ornithorhynchus anatinus*; Trivir, *Trichoderma virens*, PDB 7N7I; Nemvec, *Nematostella vectensis*, PDB 7N7H) compared with bacterial (Strapo, *Streptomyces apocyni*;

Lepkan, *Leptospira kanakyensis*) and archaeal homologs (Thesic, *Thermococcus siculi*; Methol, *Methanomethylovorans hollandica*). (**E**) Amino acid sequence alignment of C-termini of eukaryotic Ncs6/Ctu1 proteins (Saccer, *Saccharomyces cerevisiae*; Homsap, *Homo sapiens*; Danrer, *Danio rerio*; Aratha, *Arabidopsis thaliana*) with archaeal homologs (Pyrhor, *Pyrococcus horikoshii*, PDB 5MKQ; Metmar, *Methanococcus maripaludis* S2, PDB 6SCY; Halvol, *Haloferax volcanii* DS2; Themar, *Thermotoga maritima* MSB8). (**F**) Amino acid sequence alignment of C-termini of fungal Nit-6/NiiA nitrite reductases (Neucra, *Neurospora crassa* OR74A; Aspnid, *Aspergillus nidulans* FGSC A4; Magory, *Magnaporthe oryzae* 70-15; Talsti, *Talaromyces stipitatus* ATCC 10500) and their archaeal Nir1 homologs (Blamar, *Blastopirellula marina*; Nitbac. *Nitrospirales* bacterium, HIB54509.1; Brevol, *Bremerella volcania*; Gimpan, *Gimesia panareensis*). (**G**) Amino acid sequence alignment of C-termini of fungal Apd1 (cytosolic) and Aim32 (mitochondrial) homologs (Saccer, *Saccharomyces cerevisiae*; Yarlip, *Yarrowia lipolytica*; Picpas, *Pichia pastoris*; Candub, *Candida dublinensis*). (**H**) Amino acid sequence alignment of C-termini of Neverland, eukaryotic Rieske-type hydroxylases (Dromel, *Drosophila melanogaster*; Xenlae, *Xenopus laevis*; Danrer, *Danio rerio*; Bommor, *Bombyx mori*) and bacterial ketosteroid hydroxylase homologs (Nosspe, *Nostoc sp*. NIES-4103; Mycspe, *Mycobacterium sp*. Aquia_213; Halmar, *Halioglobus maricola*; Sorcel, *Sorangium cellulosum*). (**I**) Amino acid sequence alignment of C-termini of Rieske-centre containing fungal choline monoxygenases (Schsti, *Scheffersomyces stipitis* CBS 6054; Pickud, *Pichia kudriavzevii*; Canten, *Candida tenuis* ATCC 10573; Clalus, *Clavispora lusitaniae* ATCC 42720) with plant plastidic (Spiole, *Spinacia oleracea*; Glymax, *Glycine max*) and bacterial homologs (Silpal, *Silvanigrella paludirubra*; Rueare, *Ruegeria arenilitoris*).

A

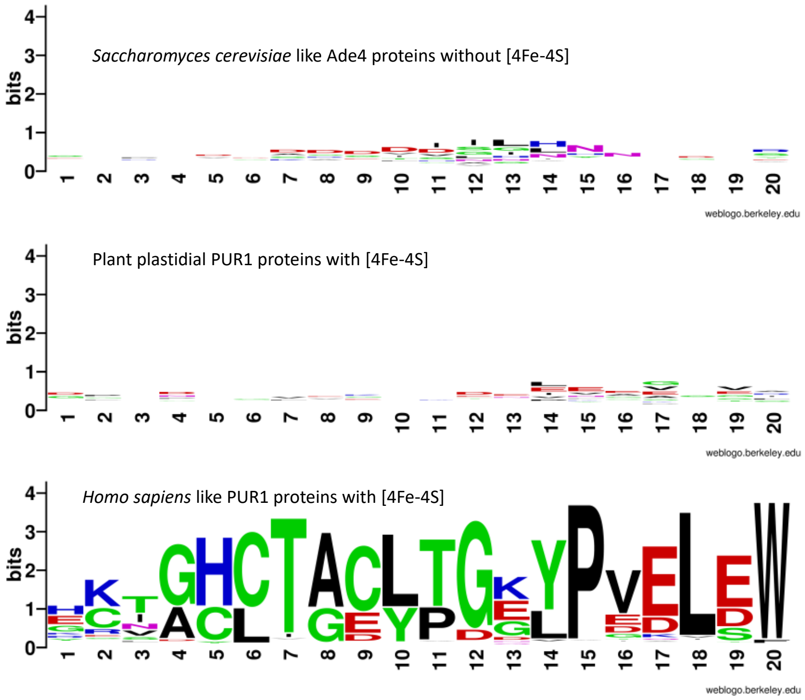

B

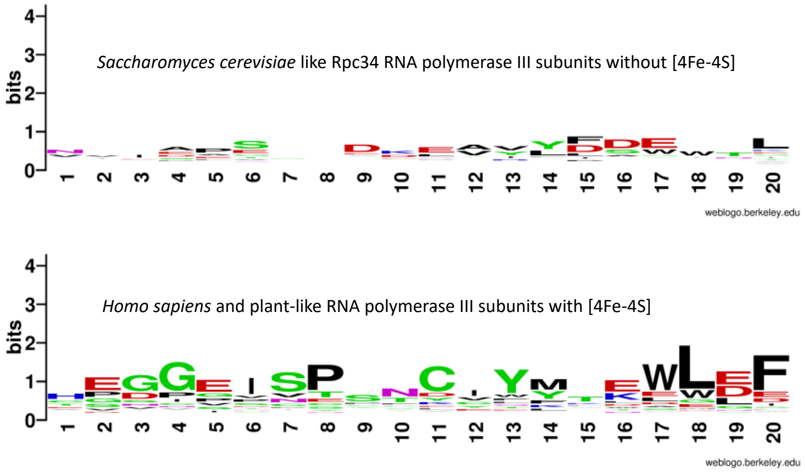

Fig. S3. Conservation of a C-terminal W/F correlates with conservation of Fe-S cluster binding ligands. (**A**). Absence (top two Weblogos) and presence (bottom) of a C-terminal W or F residue correlates with the absence and presence, respectively, of Fe-S cluster binding signatures in eukaryotic glutamine phosphoribosylpyrophosphate amidotransferases (PUR1). Weblogo depictions for the C-terminal 20 amino acids of 570 yeast-like Ade4 proteins, which do not bind a [4Fe-4S] cluster, of 205 plant PUR1 proteins, which due to their plastidial localization lack a C-terminal W/F signature, and of 468 human-like PUR1 proteins, which based on conservation of 4 cysteine residues bind a [4Fe-4S] cluster. Sequences were collected via OrthoDB (19) and sorted according to their amino acid sequence identities with yeast, human and plant PUR1/Ade4 proteins and manually annotated for the Fe-S binding ligands. (**B**). Absence (top) and presence (bottom) of a C-terminal W or F residue correlates with the absence and presence, respectively, of Fe-S cluster binding signatures in eukaryotic RNA polymerases. WebLogo depictions for the C-terminal 20

amino acids of 131 yeast-like (Rpc34) RNA polymerase III subunits, which do not bind a [4Fe-4S] cluster and of 984 human-like (POLR3F) RNA polymerase III subunits, which based on conservation of 4 cysteine residues bind a [4Fe-4S] cluster. Sequences were collected via OrthoDB. The presence of a cysteine residue corresponding to Cys307 in the human sequence in a multiple amino acid sequence alignment was used to define sequences predicted to bind a [4Fe-4S] cluster.

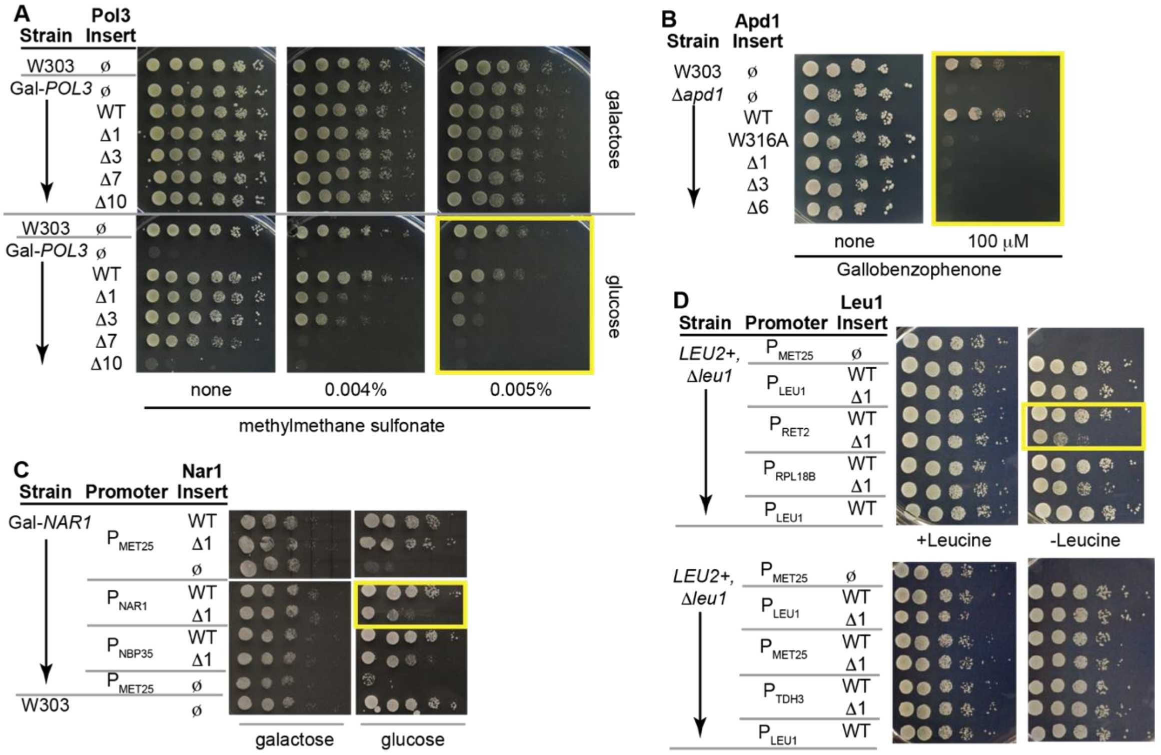

Fig. S4. Non-cropped images of droptests shown in Figure 2A-D (yellow boxes illustrate data in main text) along with controls showing growth under permissive conditions including: in the presence of glucose (**A** and **C**); in the presence of leucine (**D**); or in the absence of gallobenzophenone (**B**). For Nar1 (C) the strong *MET25* or the weaker *NBP35* and *NAR1* promoters were used to drive expression of the WT and Δ1 inserts. For Leu1 (D) and Nar1 (C), the weak (*RET2, NBP35* and *RPL18B*), moderate (*LEU1*) and strong (*MET25* and *TDH3*) promoters were used. In both cases, the stronger promoters can mask the growth defect observed for the Δ1 variants in comparison to the weaker promoters.

A

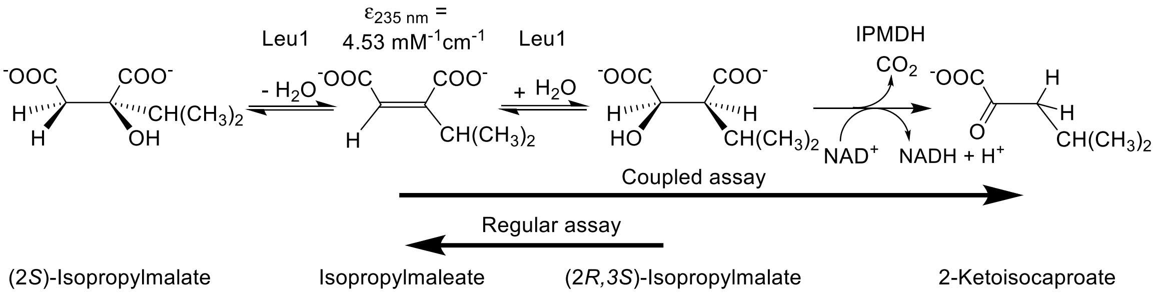

B

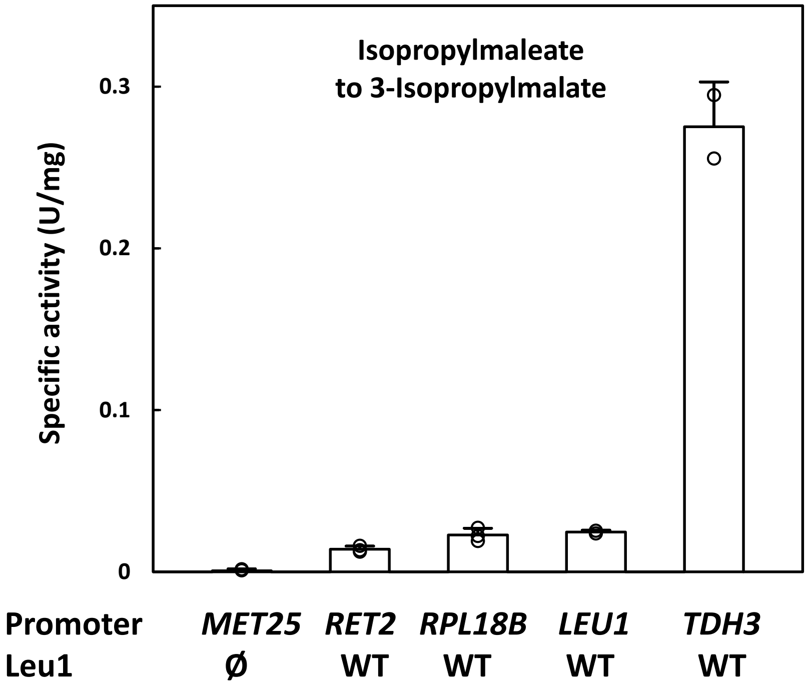

Fig. S5. (**A**) The regular (9) and coupled isopropylmalate isomerase assay. (**B**) Bar graph with specific activities of isopropylmalate isomerase in cell extracts from Δ*leu1*/*LEU2*$^+$ cells transformed with pRS416 plasmids with *LEU1* under control of indicated promoters. Cells were grown in SC glucose medium including leucine. A coupled assay (3-isopropylmalate formation from isopropylmaleate as detected by *E. coli* isopropylmalate dehydrogenase (IPMDH) dependent NADH formation was used to quantify the isopropylmalate isomerase activity in cell extracts with *RET2*, *RPL18B*, *LEU1* and *TDH3* promoter driven *LEU1* constructs. Under these growth conditions the use of a *MET25* promoter for *LEU1* expression results in 70±6 % (n=3) of the specific activity in the regular assay in comparison with the *TDH3* promoter. In the regular assay a specific activity of 0.24-0.32 U/mg (Fig. 2 and 3) or 0.45-0.50 U/mg (Fig. S7) is measured in the Δ*leu1* yeast strain with *LEU1*- or *TDH3*-driven Leu1 expression, respectively.

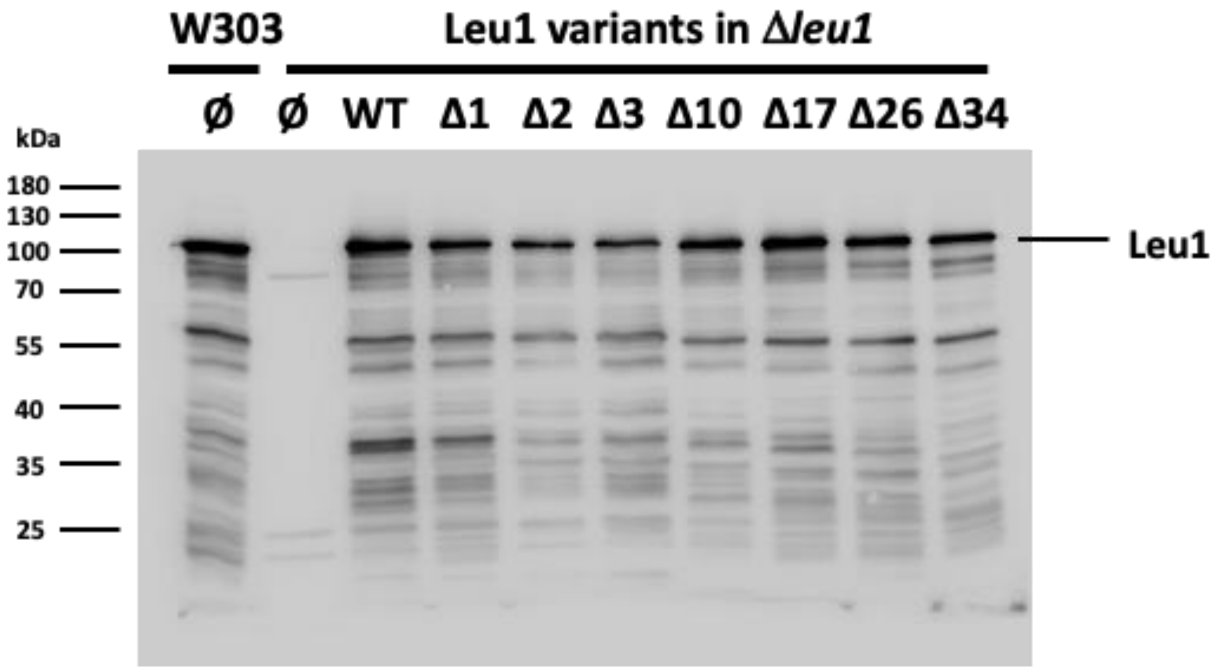

Fig. S6. Western blot of cell free extracts of the indicated yeast strains transformed with pRS416 plasmids expressing wild type (WT) Leu1 and its C-terminally truncated variants from its natural promoter (corresponding to Fig. 2E). Rabbit polyclonal antibodies raised against purified yeast Leu1 were used.

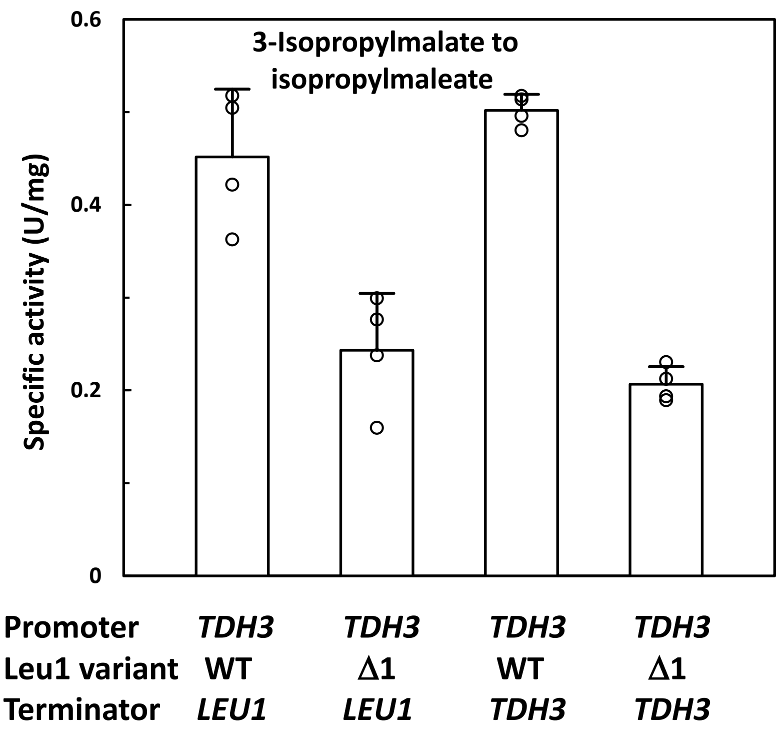

Fig. S7. The loss of activity upon removal of W779 is not due to regulatory phenomena related to *LEU1* promoter or terminator regions. Specific activities (using the regular assay) of wild-type and the Δ1 truncation of yeast Leu1 isopropylmalate isomerase in cell extracts from Δ*leu1* cells transformed with pRS416 plasmids with *LEU1* under control of the indicated promoter or terminator.

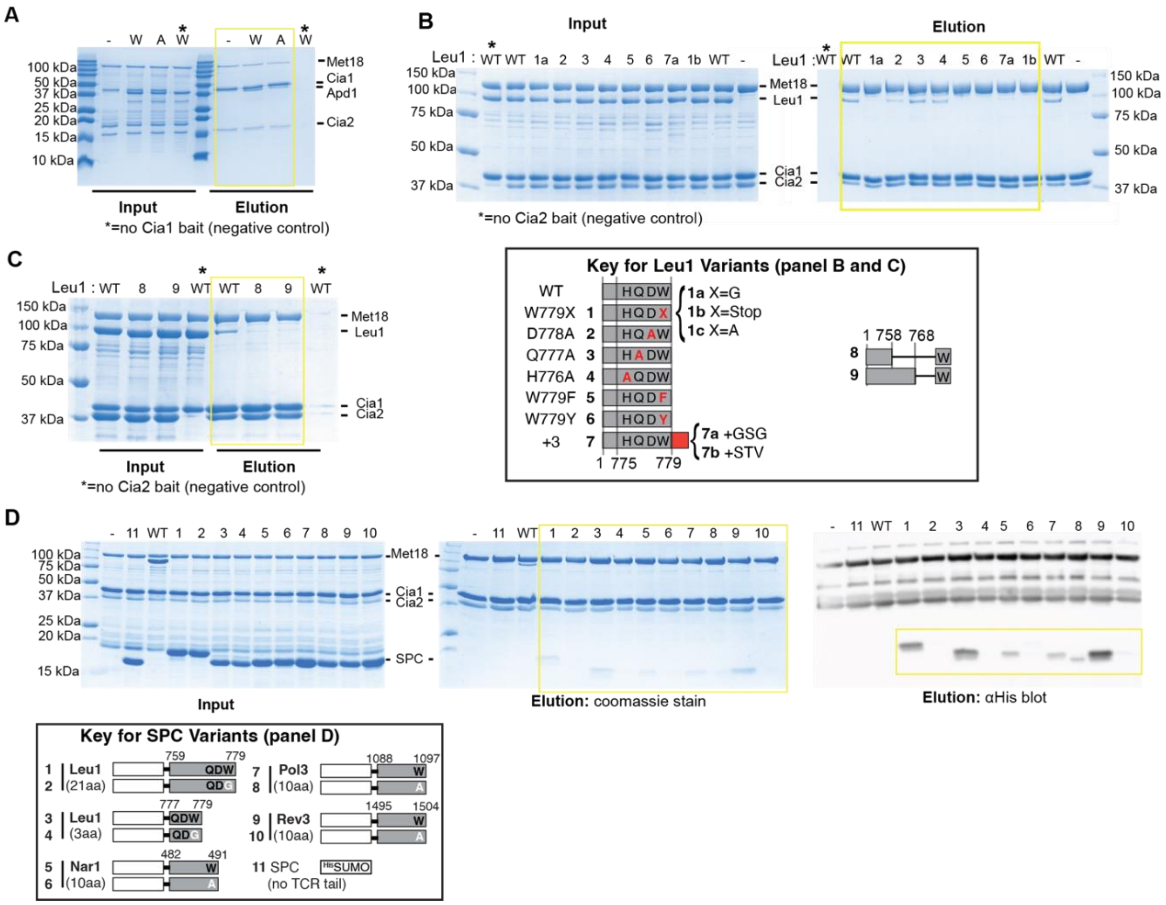

Fig. S8. Expanded copurification data for Fig. 3A (**A**), Fig. 3C (**B**), Fig. 3F (**C**), and Fig. 4A (**D**). For each panel, SDS-PAGE gels (A-C) and Western Blot (C) from copurification input and elution samples are shown with data appearing in main text boxed in yellow. Numbers across the top of each gel indicate the Leu1 or SPC-variant (see keys included in figure) included in the copurification. In all panels, a positive control with the full-length, wild-type (WT) Leu1 or Apd1 protein is also shown. Panels A - C have a negative control (*) in which the ^{Strep}Cia1 (A) or ^{DT}Cia2 (B-C) bait was omitted. Additional controls in Panels A, B and D contain only the CTC, but no TCR-tail protein (-). The higher molecular weight bands in the Western blot correspond to the CTC subunits, which all have a His-tag to facilitate their purification.

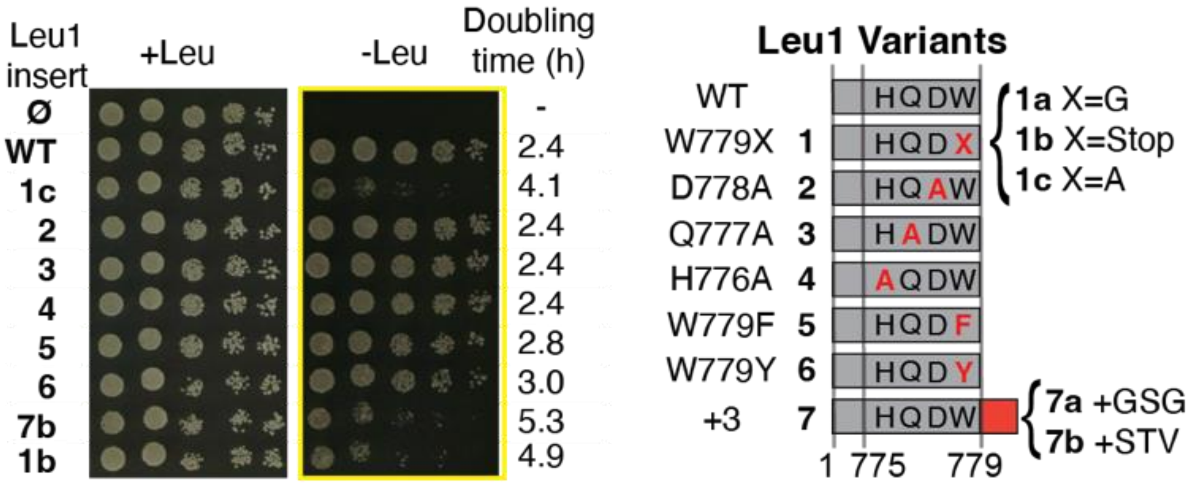

Fig. S9. Full spot tests corresponding to Fig. 3D (yellow box). The left panel is a control plate onto which the same cell suspensions were spotted onto solid media supplemented with leucine. The doubling times measured in leucine deficient liquid medium are indicated on the right.

A

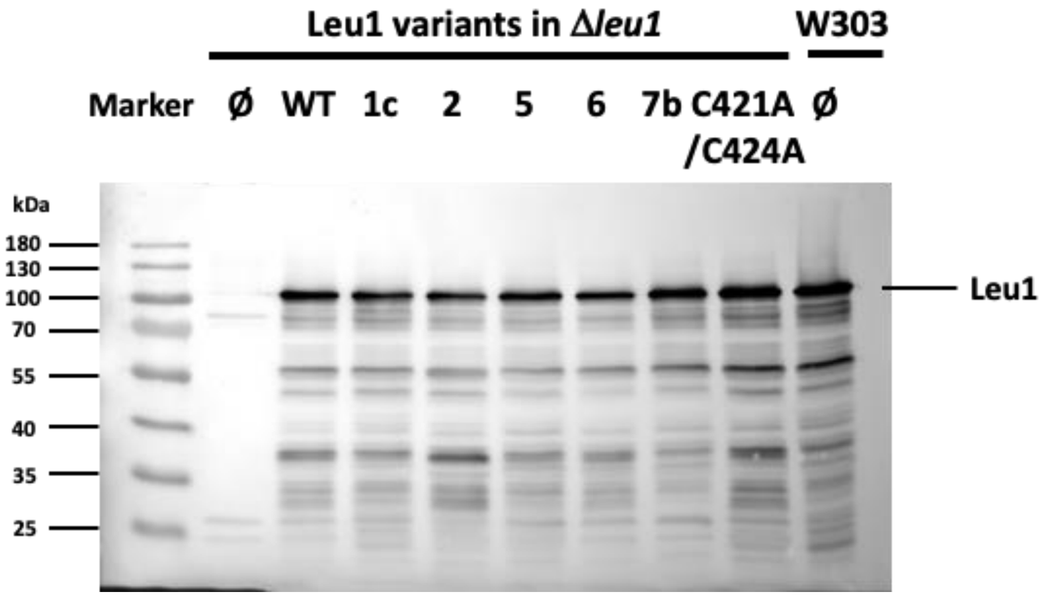

B

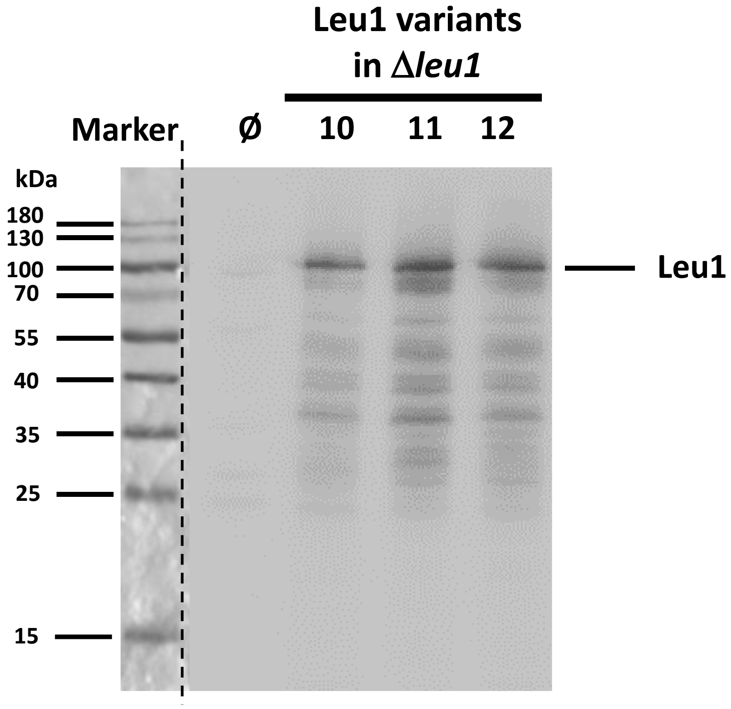

Fig. S10. Western blots of cell free extracts of the indicated yeast strains transformed with 416 plasmids expressing wild type (WT) Leu1 and its variants from its natural promoter. Empty vector, Ø. (**A**) for Fig. 3E. (**B**) for Fig. 3G. Rabbit polyclonal antibodies raised against purified yeast Leu1 were used. The protein marker in (A) was merged on top of the chemiluminescence image by the Intas ChemoStar Touch imager software, for (B) the photograph (left of the dotted line) and chemiluminescence image were manually merged.

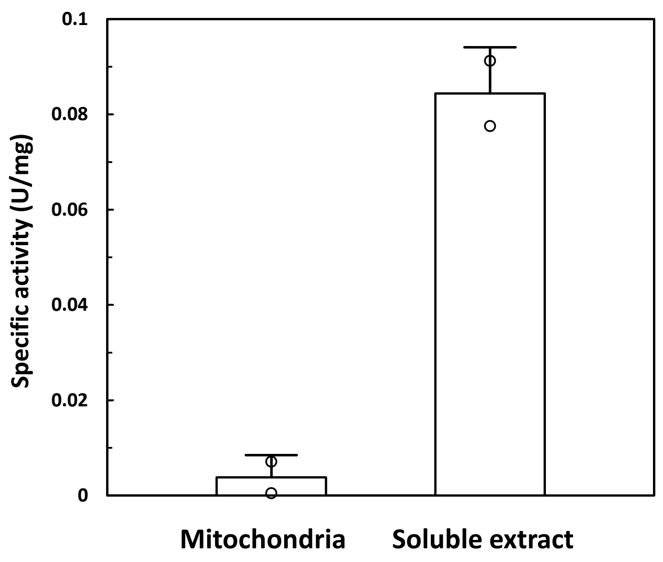

Fig. S11. Fractionation of yeast expressing the engineered *E. coli* LeuCD with yeast C-terminal sequence demonstrates that the activity in the cell extract is cytosolic. The succinate dehydrogenase specific activity of the two mitochondrial preparations was 0.25 and 0.31 U/mg.

A

```
Homsap_Elp4     -------PLYKDYHGLIHIRQIPRLNNLICDE----SDVKDLAFKLK-RKLFTI   380
Danrer_Elp4     -------PLYKDYHGLLYVRQIPRLNCLTSEV----PDTKDLAFKLK-RKQFTI   360
Schpom_Elp4     -------GLVTQPLGLFRIHKLPLALPFTNHANS--NEAGDLSFTVS-KRRFTI   329
Saccer_Elp4     RVYKS--QPGKIQHGLVHILKLPVFTDRGEMR----VLKSEWAFKNG-RKKFEI   413
Aspnid_Elp4     --SGAATSHEEPPQGMLKTHRLPVLHERGGGSDQN--IGQDWAFTLS-RRKFEI   360
Neucra_Elp4     PPSRSDPKANEQPQGMFKVYTLPVYHEKGGGGAESGQFRENLSFSLSASKGLVI   362
Dromel_Elp4     -------PAFKEYSGLLHLHKMSAINTLAVHM----PETPDLAFKLR-RKKFII   402
Aratha_Elp4     L-----LTGYKDINGFLNIHKVARINTQVPVI----LEAKTFSMSLK-KRRFLA   323
                              *:.    :                    ::.    : :

Homsap_Elp4     ER-LHLPPDLSDTVSRSSKMDLAESAKRLGPGCGMMAGGKKHLDF   424
Danrer_Elp4     ER-LHLPPDLSETVSRVSKADL-------AAGCASTATGNKHLHF   397
Schpom_Elp4     EP-WVLPPLDDEQKDTK-----------I-SNTNPQKQPVKSLDF   361
Saccer_Elp4     EQ-WGIPVDDAEGSAASEQSHSHSHSDEI-SHNIPAKKTKISLDY   456
Aspnid_Elp4     KP-FSLPPAEGDQEAQN-----HGQADK-----M---PKKEDLEF   391
Neucra_Elp4     KP-YSLPPMLEDEQEK---------PSA-----A---PKKDGLDF   389
Dromel_Elp4     EK-FHLPPELQESSAKPDNCI---------SGLLSNSNATASLDF   437
Aratha_Elp4     LECLNQAPV-DGSSGTSY---------GTSGSCS---SKSGALDF   355
                                                          *.:
```

B

```
Homsap_Pri1     VPIDLQKVDQFDPFTVPTISFICRELDAISTNEEE----K-EENEAESDVKHRT   380
Xenlae_Pri1     VPIDCKKLDQFDPFSVPTISLICSELDNVSKKEED----EDSAGEGEPEAKKRT   380
Danrer_Pri1     VPIDLKELDTFDPFEVPTISLICRELEKPRADEAEDEDVKDKENEQEAAERRRI   385
Saccer_Pri1     VPIDES----FAPEKAPKLIDLQTEME-------------------------KN   364
Asgard_PriS     I--VIKDIRNFYPDSAPTIWESLM------------------------------   325
Heimd1_PriS     CMVDIKKVHSFFPDDAPTIWEKFS------------------------------   341
Heimd2_PriS     RILDFEDFFDFNPEDEPSIFSDIEEQR---------------------------   330
Archae_PriS     QIVDKPDK--FQPHKDGLHFLEVIGKNKED------FNLK--------------   349
                          * *

Homsap_Pri1     RDYKKTSLAPYVKVFEHFLENLDKSRKGELLKKSDLQK-----DF   420
Xenlae_Pri1     RDYKRTSLAPYIKVFEQFLDKLDQSRKGELLNKSDLKK-----EF   420
Danrer_Pri1     RDYKRTSLAKYVKVLDRFLDSMARSRKGEMLKKSDLQK-----DF   425
Saccer_Pri1     NDVSLTALQPFINQFQAYVSSLLKNELGSVKREREDDDEPASLDF   409
Asgard_PriS     ---------------------------------------------   325
Heimd1_PriS     ---------------------------------------------   341
Heimd2_PriS     ---------------------------------------------   330
Archae_PriS     ---------------------------------------------   349
```

Fig. S12. Amino acid sequence alignment for TCR-containing Elp4 and Pri1 non-Fe-S subunits of elongator and primase, respectively. (**A**) Elp4 subunits (Homsap, *Homo sapiens*; Danrer, *Danio rerio*; Schpom, *Schizosaccharomyces pombe*; Saccer, *Saccharomyces cerevisiae;* Aspnid, *Aspergillus nidulans*; Neucra, *Neurospora crassa*; Dromel, *Drosophila melanogaster*; Aratha, *Arabidopsis thaliana*) end in a TCR signal (red). Eubacteria lack Elongator complexes. Archaea only have Elp3, but not Elp4 and the other subunits. The Elongator complex of *A. thaliana* is cytosolic. (**B**) Primase small subunits from Fungi and animals (bold) have a 40-60 amino acid extension and end in a TCR signal (red). In contrast, the primase small subunits from Asgard/Heimdall Archaeota, which are the closest non-eukaryotic homologs, do not have this extension. Homsap, *Homo sapiens*; Xenlae, *Xenopus laevis*; Danrer, *Danio rerio*; Saccer, *Saccharomyces cerevisiae*; Asgard_PriS, NPD89472.1; Heimd1_PriS, MBN1330204.1; Heimd2_PriS, PWI48945.1; Archae_PriS, NDB28160.1).

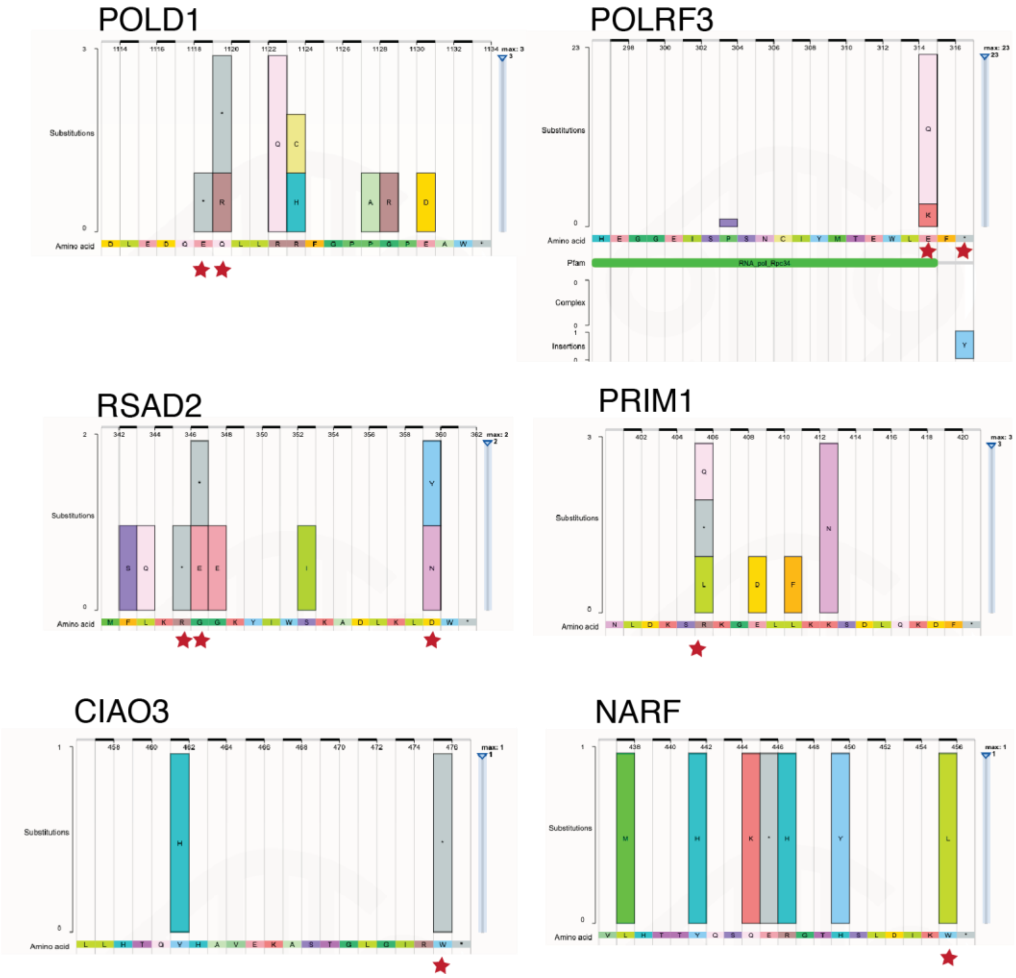

Fig. S13. Somatic mutations associated with cancer for cytosolic and nuclear Fe-S proteins carrying a TCR. For six proteins mutations occur, which lead to loss of the TCR functionality (substitution in the tripeptide, of the stopcodon or C-terminal truncation of up to 20 amino acids; red star). The outputs of the COSMIC database (https://cancer.sanger.ac.uk/cosmic) depict the mutations in form of a histogram at the level of amino acids (numbering at the top, amino acid in the wild type protein indicated at the bottom). Grey rectangles correspond to nonsense mutations.

Table S1. Overview of failed expression of active Fe-S enzymes in heterologous hosts for biotechnological purposes.

Pathway	Product	Reference
Entner-Doudoroff	Ethanol	(20)
Entner-Doudoroff	Isobutanol	(19)
Mosaik	Isobutanol	(21)
Ehrlich	Isobutanol	(22)
Dahms (D-Xylose oxidation)	Ethyleneglycol	(23)
Weimberg	2-Ketoglutarate	(24)
2-C-Methyl-D-erythritol-4-phosphate (MEP)	Isoprenoids	(25)
2-C-Methyl-D-erythritol-4-phosphate (MEP)	Isoprenoids	(26)
1-Deoxy-D-xylulose-5-phosphate (DXP)	Isoprenoids	(27)

Table S2. Proteomic data and structural data for TCR-containing proteins demonstrating that the TCR motif is not processed after Fe-S protein maturation.

Source[1]	Protein Name	Occurrence[2]	Coverage (%)[1]	Calc. mass[3]	C-terminal peptide(s) observed and notes
Homo sapiens	POLD1[4]	-	-	-	Up to W1107 in structure
	POLD1	170	98.9	956.4	(K)(DLE)(DQEQLLR)(R)FGPPGP EAW
	PUR1	72	100	934.4	(E)(K)(SGH)(CTACL)(TG)(K)YPVE LEW
	NARF	15	89.2	204.1	(R)(G)THSLDIKW, non-tryptic digest
	PRIM1	4	97.3	280.1	(LLKK)SDLQKDF, non-tryptic and incomplete tryptic digests
	ELP4	3	91.5	530.2	(DLAE)SAKRLGPGCGMMAGGKK HLDF, non-tryptic digest
	CIAO3	2	98.3	204.1	ASTGLGIRW, incomplete tryptic digest
	LTO1	2	95.6	966.5	ISAEGSGLSF
	POLR3F	1	93.9	3561.5	APCGLCPVFDDCHEGGEISPSN CIYMTEWLEF
	CTU1	1	88.5	533.3	PARPPASKAVPTF, non-tryptic digest
	REV3L[5]	0	52.5	762.4	Too small, QLLDQF
	RSAD2[5]	0	62.0	432.2	Too small, LDW
Saccharomyces cerevisiae	Leu1	27	100	683.3	(K)AVTTTFDKVHQDW, incomplete tryptic digest
	Pri1	21	89.9	1251.5	(R)(ER)EDDDEPASLDF
	Nar1	3	78.8	962.5	DLVSVGSTW
	Pol3[6]	-	-	-	Up to W1097 in structure
	Pol3	1	81.7	204.1	VEQLSKW, incomplete tryptic digest
	Lto1[7]	1	83.3	860.4	QNQAQSW
	Rev3[5]	0	12.9	1188.6	EEALISLNDW
	Apd1[5]	0	94.3	582.2	Too small, YQSW
	Ncs6[5]	0	72.4	365.2	Too small, LSF
	Elp4[5]	0	74.3	609.3	Too small, ISLDY

[1] Mass spectrometric data were collected with the PeptideAtlas online tool (28).
[2] The number of experiments for which the C-terminal peptide was detected in the canonical isoform.
[3]The calculated masses (monoisotopic) are for the C-terminal tryptic peptide.
[4] PolD1 was expressed in insect cells and its structure was determined by cryoEM (PDB 61SM).
[5] Failure to detect C-terminal peptides explained by the small size of the C-terminal peptide (<7 amino acids) or the moderate coverage due to the low cellular protein abundance.
[6] Pol3 was expressed in yeast and its structure was determined by X-ray crystallography (PDB 6P1H)
[7] Lto1 coverage corrected to take the assignment of the N-terminal sequence (MDF) into account which is supported by the absence of peptides corresponding to the erroneously assigned MVR N-terminus (29).

Table S3. Mutagenesis primers for plasmids relating to *in vitro* interaction studies.

Construct	Primer	Sequence (5'-3')
Leu1 W779G	Forward	GAT AAA GTC CAC CAG GAT GGG TAG CTC GAG GAT CCG
	Reverse	CGG ATC CTC GAG CTA CCC ATC CTG GTG GAC TTT ATC
Leu1D778A	Forward	AGT CCA CCA GGC GTG GTA GAT GG
	Reverse	TTA TCG AAA GTA GTT GTA AC
Leu1 Q777A	Forward	GTC CAC GCG GAT TGG TAG C
	Reverse	TTT ATC GAA AGT AGT TGT AAC A
Leu1H776A	Forward	AAA GTC GCG CAG GAT TGG TAG
	Reverse	ATC GAA AGT AGT TGT AAC AGC T
Leu1W779F	Forward	C CAG GAT TTC TAG CTC GAG G
	Reverse	TGG ACT TTA TCG AAA GTA GTT G
Leu1W779Y	Forward	CCA GGA TTA CTA GCT CGA GG
	Reverse	TGG ACT TTA TCG AAA GTA GTT G
Leu1+GSG	Forward	GGG GTA GCT CGA GGA TCC GGC
	Reverse	GAC CCC CAA TCC TGG TGG ACT TTA
Leu1-Δ1	Forward	CGC TGA GTA ATA ACT AGC ATA AC
	Reverse	GTG GCA GCA GCC AAC TCA G
Leu1$_{\Delta 759\text{-}778}$	Forward	TGG TAG CTC GAG GAT CCG
	Reverse	TTT TAA TAA TTT TGA TCC ACC TTC
Leu1$_{\Delta 769\text{-}778}$	Forward	TGG TAG CTC GAG GAT CCG
	Reverse	AAC AGC TTT TCT TTT TGG AAC G
SPC-Leu1$_{759\text{-}779}$	Forward	CTA CTT TCG ATA AAG TCC ACC AGG ATT GGT AGA CTC GAG CCC GGG TGA CT
	Reverse	TTG TAA CAG CTT TTC TTT TTG GAA CGT TGT CAA AAG CTT GTC GAC GGA GCT C
SPC-Leu1$_{759\text{-}779}$ W779G	Forward	CCA GGA TGG ATA GAC TCG A
	Reverse	TGG ACT TTA TCG AAA GTA GTT G
SPC-QDW	Forward	CAG GAT TGG TAG ACT CGA G
	Reverse	AGC TTG TCG ACG GAG CTC
SPC-QDG	Forward	CAG GAT GGG TAG ACT CGA G
	Reverse	AGC TTG TCG ACG GAG CTC
SPC-Nar1$_{482\text{-}491}$ W491A	Forward	C ACG ACG GCG TAA CTC GAG
	Reverse	CC AAC GCT AAC CAG ATC
SPC-Pol3$_{1088\text{-}1097}$ W1097A	Forward	CAG CTG AGC AAA GCG TAA CTC
	Reverse	CTC CAC TTT TTC TTG GCG
SPC-Rev3$_{1495\text{-}1504}$	Forward	TCT CTA AAT GAT TGG TAG ACT CGA GCC CGG GTG
	Reverse	TAT TAA TGC TTC TTC AAA AGC TTG TCG ACG GAG
SPC-Rev3$_{1495\text{-}1504}$ W1504A	Forward	GAT GCG TAG ACT CGA GCC
	Reverse	ATT TAG AGA TAT TAA TGC TTC TTC
^{His}Apd1 W316A	Forward	GGG GAT ACC CAG CTT TCT TGT AC
	Reverse	GGG AAT TGC CAT GTT ACG CGC TT

Table S4. Yeast strains.

Strain	Genotype	Reference
Gal-*NFS1*	W303-1A pNFS1::HIS3-pGAL1-10	(30)
Gal-*CFD1*	W303-1A pCFD1::HIS3-pGAL1-10	(31)
Gal-*CIA1*	W303-1A pCIA1::HIS3-pGAL1-10	(32)
Gal-*NAR1*	W303-1A pNAR1::HIS3-pGAL1-10	(33)
Gal-*POL3*	W303-1A pPOL3::HIS3-pGALL	This work
Δ*leu1*	leu1::natNT2	This work
LEU2^{+}/Δ*leu1*	W303-1A leu2-3,112::leu2, leu1::natNT2	This work
Δ*apd1*	apd1::natNT2	(2)
Gal-*NFS1*/Δ*leu1*	Gal-*NFS1*, leu1::natNT2	This work
Gal-*NAR1*/Δ*leu1*	Gal-*NAR1*, leu1::natNT2	This work
Gal-*CIA1*/Δ*leu1*	Gal-*CIA1*, leu1::natNT2	This work
Gal-*CFD1*/Δ*leu1*	Gal-*CFD1*, leu1::natNT2	This work

Table S5: Primers for homologous recombination.

Strain	Primer	Primer Sequence (5'-3')
Gal-*POL3*	Pol3_S1	TAT TGA GCA CTT GCT ATT AAG CAT TAA TCT TTA TAC ATA TAC GCA CAG CAC GTA CGC TGC AGG TCG AC
	Pol3_S4	TCG TCA TCG ATC TTC ACA TCA ACC ATG GGA AGG GAT CTT TTT TCA CTC ATC GAT GAA TTC TCT GTC G
Δ*leu1*	Leu1_S1	GAA ATT GAC AGT TTT TGT CGC TAT CGA TTT TTA TTA TTT GCT GTT TTA AAT CAT GCG TAC GCT GCA GGT CGA C
	Leu1_S2	CGC TGA GAC ACA TGT TAT TGA CGC CAG GTT TGG ACG TTG TTT TTC ACT GTC TAA TCG ATG AAT TCG AGC TCG
LEU2$^+$	A forward	ATG TCT GCC CCT AAG AAG ATC GTC GTT TTG C
	A reverse	TAC CGG TAC CCC ATT TAG GAC CAC CCA CAG C
	B forward	CTA AAT GGG GTA CCG GTA GTG TTA GAC CTG AAC
	B reverse	TGG CAA CAA ACC CAA GGA ACC TGG GAT AAC GG
	C forward	GTT CCT TGG GTT TGT TGC CAT CTG CGT CCT TGG
	C reverse	TTA AGC AAG GAT TTT CTT AAC TTC TTC GG

Table S6. Primers used for cloning and mutagenesis. Restriction sites are in bold.

Construct	Sequence (5'-3')
Pol3_SacI	AAG CTG **GAG CTC** AAT TTC GAC GCC AGT ATC C
Pol3_EcoRI	GAT ATC **GAA TTC** TTA CCA TTT GCT TAA TTG TTC TAC TTT CTC C
Pol3_D1_for	CAA TTA AGC AAA TGA TAA GAG TTC GAT ATC AAG C
Pol3_D1_rev	CGA ACT CTT ATC ATT TGC TTA ATT GTT CTA CTT TC
Pol3_D3_for	GAA CAA TTA TGA AAA TGG TAA GAA TTC GAT ATC
Pol3_D3_rev	CCA TTT TCA TAA TTG TTC TAC TTT CTC CTG C
Pol3_D7_for	GGA GAA ATG AGA ACA ATT AAG CAA ATG GTA AG
Pol3_D7_rev	GTT CTC ATT TCT CCT GCA GCT CTT TTT TAA CC
Pol3_D10_for	GAG CTG TAG GAG AAA GTA GAA CAA TTA AGC
Pol3_D10_rev	CTT TCT CCT ACA GCT CTT TTT TAA CCT TAA CC
Apd1_NP_SacI	ATG GTA **GAG CTC** CTG CTG GGC TTG TAA TCA TTG
Apd1_NT_KpnI	TGG TA**G GTA CC**T AGC GGT GGC GGT AGA AAT CTG
Apd1_W316A_for	CAA AGC GCG TAA GTC GAC CTC GAG GTT AAT TC
Apd1_W316A_rev	CGA GGT CGA CTT ACG CGC TTT GAT ATT TCT TA
Apd1_D1_for	CAA AGC TGA TAA GTC GAC CTC GAG GTT AAT TC
Apd1_D1_rev	CGA GGT CGA CTT ATC AGC TTT GAT ATT TCT TA
Apd1_D3_for	GAA ATA TTG AAG CTG ATA AGT CGA CCT CGA GG
Apd1_D3_rev	GAC TTA TCA GCT TCA ATA TTT CTT AAT ACA GCG
Apd1_D6_for	GCT GTA TTT AGT AAT AGC AAA GCT GGT AAG TCG
Apd1_D6_rev	CTT TGC TAT TAC TAA ATA CAG CGA ACT TTT TCG
Nar1_NP_SacI	ATG GTA **GAG CTC** TTT TTA AAA CAA TTT GTG GTG CTC G
Nar1_NT_KpnI	ATG GTA **GGT ACC** TTG CCT TAT GTG GTT TTC TAT TC
Nar1_SpeI	ATG GTA **ACT AGT** ATG AGT GCT CTA CTG TCC GAG TCT G
PromNpb35_SacI	ATG GTA **GAG CTC** CCG TAA AGA TTC GCA AAT CAT TG
PromNbp35_SpeI	ATG GTA **ACT AGT** CTT GGA TAT CAC TTA TGT ATA CTG
Nar1_D1_for	GAG CAC CTG ACT AGT AAG GTG TAG TTT TGC ACC
Nar1_D1_rev	CCT TAC TAG TCA GGT GCT CCC AAC AGA GAC GAG
Leu1_SalI	GAA GCT **GTC GAC** GTG ATT CCA ACT ATT AC
Leu1_NgoMIV	ATG GTA **GCC GGC** ACT CAT CGA TCA CAG G
Leu1_SacI	TGG TA**G AGC TC**A AGA TAC ACA CTT ACA TGA ACG
Leu1_SalI	AAT CAC **GTC GAC** AGC TTC AAT GTT GAT TTC G
Leu1_SpeI_for	ATT TGC TGT T**AC TAG T**CA TGG TTT ACA CTC CAT CC
Leu1_SpeI_rev	CAT G**AC TAG T**AA CAG CAA ATA ATA AAA ATC GAT AGC G
Met25_SacI_for	ATG GTA **GAG CTC** CGG ATG CAA GGG
Met25_SpeI_rev	ATG GTA **ACT AGT** TCT AGA GTA TGG ATG G
TDH3_SacI_for	ATG GTA **GAG CTC** AGT TTA TCA TTA TCA ATA CTG C
TDH3_SpeI_rev	ATG GTA **ACT AGT** TCT AGA ATC CGT CGA AAC TAA G
Esccol_LeuB_BamHI	ATG GTA **GGA TCC** CAT GTC GAA GAA TTA CCA TAT TGC CG
Esccol_LeuB_HindIII	ATG GTA **AAG CTT** TAC ACC CCT TCT GCT ACA TAG C
Leu1_mutXhoI_for	GAT TGG TAG **CTC GAG** AAA AAC AAC GTC CAA ACC
Leu1_mutXhoI_rev	GTT GTT TTT **CTC GAG** CTA CCA ATC CTG GTG GAC
Leu1_mutBamHI_for	CAA CCC TG**G GAT CC**A TGA GTG CGC CGG CTT TCC C
Leu1_mutBamHI_rev	CTC AT**G GAT CC**C AGG GTT GAT TTA ACA TTT AAG C
TDH3term_XhoI	ATG GTA **CTC GAG** ATT TAC TTT AAA TCT TGC
TDH3term_BamHI	ATG GTA **GGA TCC** GCG CGT ACA TTT AAT TTT CAA CG
Leu1_D1_TDHter_for	GGA TTG ATA GCT CGA GTG AAT TTA CTT TAA ATC
Leu1_D1_TDHter_rev	CAC TCG AGC TAT CAA TCC TGG TGG ACT TTA TCG
RET2_SacI_for	ATG GTA **GAG CTC** AGG GCA AGT AAA GTG TTT GAT TGG
RET2_SpeI_rev	ATG GTA **ACT AGT** GTG TAT TTC TTT TGA TGG AGC
RPL18B_SacI_for	ATG GTA **GAG CTC** TAA CAA TCT CTT TGC AAA TGT GGG
RPL18B_SpeI_rev	ATG GTA **ACT AGT** TTG TTT TTT GTT TTC TTC TAA TTG

FBAprom_SacI	ATG GTA **GAG CTC** ACT GGT AGA GAG CGA CTT TG
FBAprom_SpeI	ATG GTA **ACT AGT** TTG AAT ATG TAT TAC TTG GTT ATG G
FBAterm_XhoI	ATG GTA **CTC GAG** TTA ATT CAA ATT AAT TGA TAT AG
FBAterm_KpnI	ATG GTA **GGT ACC** TTT AAC GTA TAG ACT TCT AAT ATA TTT C
Leu1_CT_EcoRI	ATG GTA **GAA TTC** ATG AAG CCA TTT TTG ACT TTG G
Leu1_CT_BamHI	ATG GTA **GGA TCC** TAC CAA TCC TGG TGG ACT TTA
LeuC_SpeI	ATG GT**A CTA GT**A TGG CTA AGA CGT TAT ACG AA
LeuC_XhoI	ATG GTA **CTC GAG** TTA TTT AAT GTT GCG AAT GTC GGC
LeuD_SpeI	ATG GT**A CTA GT**A TGG CAG AGA AAT TTA TCA AAC ACA C
LeuD_XhoI	ATG GTA **CTC GAG** ATT CAT AAA CGC AGG TTG TTT TGC
LeuC_Leu1_rev	CTT GAT CCT TAT ATT TAA TGT TGC GAA TGT CGG CG
LeuC_Leu1LK_for	TCG CAA CAT TAA ATA TAA GGA TCA AGA CCA AAG
Ecoli_XhoI_+10_for	ATG GTA **CTC GAG** ACT ACT TTC GAT AAA GTC CAC C
Ecoli_LeuD_XhoI_rev	TCC ACC **CTC GAG** ATT CAT AAA CGC AGG
Leu1LK_Spe_rev	ATG GTA **ACT AGT** GCT AGG TTT AGC AGG TGT ATC
SSGLinker_QDW_for	ATG GTA **CTC GAG** GGT TCT TCC GGA TCA TCG GG
SSGLinker_QDW_rev	ATG GTA **GAA TTC** TTT GCA GGA AAG TTT TGA CC
LeuD_Stop_Leu1_CTf	CGT TTA TGA ATT AAG AGG GTG GAT CAA AAT TAT
LeuD_Stop_Leu1_CTr	CCA CCC TCT TAA TTC ATA AAC GCA GGT TGT TTT
LeuD_QDW_for	GAA TCA GGA CTG GTG ATC AAA ATT ATT AAA ATT TGA CAA CG
LeuD_QDW_rev	GAT CAC CAG TCC TGA TTC ATA AAC GCA GGT TGT TTT GC
Leu1CT_mutWStop_for	TTA GTA GTC GAG CTC ATT CAA ATT AAT TGA TA
Leu1CT_mutWStop_rev	GAA TGA GCT CGA CTA CTA ATC CTG GTG GAC TTT
Leu1_mut_D1_for	ACC AGG ATT GAT AAC TAG TGA AAA ACA ACG TCC
Leu1_mut_D1_rev	TTC ACT AGT TAT CAA TCC TGG TGG ACT TTA TCG
Leu1_mut_D2_for	ACC AGT AGT ACT AGT CAG TGA AAA ACA ACG TCC
Leu1_mut_D2_rev	CTG ACT AGT ACT ACT GGT GGA CTT TAT CGA AAG
Leu1_mut_D3_for	GTC CAC TAG TAT TGG TAG ACA GTG AAA AAC AAC
Leu1_mut_D3_rev	TCT ACC AAT ACT AGT GGA CTT TAT CGA AAG TAG
Leu1_mut_D10_for	ACA TAG ACT AGT GAT AAA GTC CAC CAG GAT TGG
Leu1_mut_D10_rev	GGA CTT TAT CAC TAG TCT ATG TAA CAG CTT TTC
Leu1_mut_D17_for	AAC GTT TAA ACT AGT AAA GCT GTT ACA TGT TAC
Leu1_mut_D17_rev	CAG CTT TAC TAG TTT AAA CGT TGT CAA ATT TTA
Leu1_mut_D26_for	GTG GAT AAA AAC TAG TAA AAT TTG ACA ACG TTC
Leu1_mut_D26_rev	TTT ACT AGT TTT TAT CCA CCT TCC AAG AAT GAG
Leu1_mut_D34_for	AAG AGA GTA ATA CTA GTT CTT GGA AGG TGG ATC
Leu1_mut_D34_rev	AAG AAC TAG TAT TAC TCT CTT CTC AAA GCT TCG
Leu1_mut_+STV_for	GAT TGG TCG ACA GTG TAA AAC AAC GTC CAA ACC
Leu1_mut_+STV_rev	TAC ACT GTC GAC CAA TCC TGG TGG ACT TTA TCG
Leu1_mut_W779A_for	ACC AGG ATG CGT AAC TAG TGA AAA ACA ACG TCC
Leu1_mut_W779A_rev	TTC ACT AGT TAC GCA TCC TGG TGG ACT TTA TCG
Leu1_mut_D778A_for	ACC AGG CTT GGT AAC TAG TGA AAA ACA ACG TCC
Leu1_mut_D778A_rev	TTC ACT AGT TAC CAA GCC TGG TGG ACT TTA TCG
Leu1_mut_+GSG_for	TGG GGG TCA GGG TAG AAC AAC GTC CAA ACC TGG CG
Leu1_mut_+GSG_rev	TGT TCT ACC CTG ACC CCC AAT CCT GGT GGA CTT TAT CG
Leu1_mut_W779F_for	ACC AGG ATT TCT AAC TAG TGA AAA ACA ACG TCC
Leu1_mut_W779F_rev	TTC ACT AGT TAG AAA TCC TGG TGG ACT TTA TCG
Leu1_mut_W779Y_for	ACC AGG ATT ATT AAC TAG TGA AAA ACA ACG TCC
Leu1_mut_W779Y_rev	TTC ACT AGT TAA TAA TCC TGG TGG ACT TTA TCG
Leu1_C424A_for	CTG GTT GTT CAA TAG CTT TAG GTA TGA ACC CTG
Leu1_C424A_rev	CCT AAA GCT ATT GAA CAA CCA GCT TCT CTC CAT
Leu1_C421A/C424A_for	CTG GTG CTT CAA TAG CTT TAG GTA TGA ACC CTG

Leu1_C421A/C424A_rev	CCT AAA GCT ATT GAA GCA CCA GCT TCT CTC CAT
Leu1_D3_pET_for	GTC CAC TAG TAT TGG TAG CTC GAG GAT CCG GC
Leu1_D3_pET_rev	GCT ACC AAT ACT AGT GGA CTT TAT CGA AAG TAG
Leu1_1849_BglIIf	GCT G**AG ATC T**TG GTT GTT ACT GGT GAC AAT TTC G
Leu1_1849_BglIIr	CAG TAA CAA CCA **AGA TCT** CAG CTT CCC TCC AAG G

Table S7: Yeast plasmids. Δ refers to C-terminal truncation.

Plasmid	Promoter	Terminator	Encoded protein (variant)
416	*POL3*	*CYC*	Pol3, yeast polymerase δ δsubunit, wild type
416	*POL3*	*CYC*	Pol3, yeast polymerase δ subunit, Δ1
416	*POL3*	*CYC*	Pol3, yeast polymerase δ subunit, Δ3
416	*POL3*	*CYC*	Pol3, yeast polymerase δ subunit, Δ7
416	*POL3*	*CYC*	Pol3, yeast polymerase δ subunit, Δ10
416	*APD1*	*APD1*	Apd1, wild type
416	*APD1*	*APD1*	Apd1, W316A
416	*APD1*	*APD1*	Apd1, Δ1
416	*APD1*	*APD1*	Apd1, Δ[illegible]
416	*APD1*	*APD1*	Apd1, Δ[illegible]
416	*MET25*	*NAR1*	Nar1, wildtype
416	*MET25*	*NAR1*	Nar1, Δ1
416	*NAR1*	*NAR1*	Nar1, wildtype
416	*NAR1*	*NAR1*	Nar1, Δ1
416	*NBP35*	*NAR1*	Nar1, wildtype
416	*NBP35*	*NAR1*	Nar1, Δ1
416	*LEU1*	*LEU1*	Leu1
416	*LEU1*	*LEU1*	Leu1, SpeI site before start ATG
416	*LEU1*	*LEU1*	Leu1, SpeI site before start ATG, Δ1
416	*RET2*	*LEU1*	Leu1, SpeI site before start ATG, wildtype
416	*RET2*	*LEU1*	Leu1, SpeI site before start ATG, Δ1
416	*RPL18B*	*LEU1*	Leu1, SpeI site before start ATG, wildtype
416	*RPL18B*	*LEU1*	Leu1, SpeI site before start ATG, Δ1
416	*MET25*	*LEU1*	Leu1, SpeI site before start ATG, wildtype
416	*MET25*	*LEU1*	Leu1, SpeI site before start ATG, Δ1
416	*TDH3*	*LEU1*	Leu1, SpeI site before start ATG, wildtype
416	*TDH3*	*LEU1*	Leu1, SpeI site before start ATG, Δ1
416	*TDH3*	*TDH3*	Leu1, SpeI site before start ATG, wildtype
416	*TDH3*	*TDH3*	Leu1, SpeI site before start ATG, Δ1
416	*RET2*	*LEU1*	Leu1, SpeI site before start ATG, W779G
416	*RET2*	*LEU1*	Leu1, SpeI site before start ATG, D778A
416	*RET2*	*LEU1*	Leu1, SpeI site before start ATG, Q777A
416	*RET2*	*LEU1*	Leu1, SpeI site before start ATG, H776A
416	*RET2*	*LEU1*	Leu1, SpeI site before start ATG, W779F
416	*RET2*	*LEU1*	Leu1, SpeI site before start ATG, W779Y
416	*RET2*	*LEU1*	Leu1, SpeI site before start ATG, extended by GSG
416	*RET2*	*LEU1*	Leu1, SpeI site before start ATG, Δ1
416	*LEU1*	*LEU1*	Leu1, Δ1
416	*LEU1*	*LEU1*	Leu1, Δ2
416	*LEU1*	*LEU1*	Leu1, Δ3
416	*LEU1*	*LEU1*	Leu1, Δ10
416	*LEU1*	*LEU1*	Leu1, Δ17
416	*LEU1*	*LEU1*	Leu1, Δ26
416	*LEU1*	*LEU1*	Leu1, Δ34
416	*LEU1*	*LEU1*	Leu1, W779A
416	*LEU1*	*LEU1*	Leu1, D778A
416	*LEU1*	*LEU1*	Leu1, W779F
416	*LEU1*	*LEU1*	Leu1, W779Y
416	*LEU1*	*LEU1*	Leu1, extended by STV
416	*LEU1*	*LEU1*	Leu1, C421A/C424A
416	*TDH3*	*TDH3*	Leu1 with BglII (no amino acid change) at pos. 1847-52

416	*TDH3*	*TDH3*	Idem, but with the 19 C-terminal amino acids of yeast Leu1 replaced by the *S. pombe* Leu2 (corresponds to yeast Leu1)
416	*TDH3*	*TDH3*	Idem, but with the 19 C-terminal amino acids of yeast Leu1 replaced by the *A. nidulans* Leu1
424	*FBA1*	*FBA1*	*E. coli* LeuC in SpeI/XhoI
426	*FBA1*	*FBA1*	*E. coli* LeuD in SpeI/XhoI
426	*FBA1*	*FBA1*	Yeast Leu1 (C-terminal 30 amino acids, stopcodon)
426	*FBA1*	*FBA1*	*E. coli* LeuD and C-term. 30 amino acids of yeast Leu1
426	*FBA1*	*FBA1*	*E. coli* LeuC, yeast Leu1 linker (amino acids 481-543), *E. coli* LeuD and C-term. 30 amino acids of yeast Leu1
426	*FBA1*	*FBA1*	The above, but no Leu1 C-terminus
426	*FBA1*	*FBA1*	The above, but Δ1
426	*FBA1*	*FBA1*	The above, but only QDW as Leu1 C-terminus
426	*FBA1*	*FBA1*	The above, but only 10 C-terminal amino acids of Leu1
426	*FBA1*	*FBA1*	The above, but with LEG-$(SSG)_8$-QDW as C-terminus

Table S8: *E. coli* expression plasmids. The wild type Leu1 plasmid was described in Netz et al.(31)

Plasmid	Encoded protein (variant)
pET15b	N-terminally His-tagged Leu1, wild type
pET15b	N-terminally His-tagged Leu1, Δ3
pET15b	N-terminally His-tagged Leu1, Δ10
pET15b	N-terminally His-tagged Leu1, Δ17
pET15b	N-terminally His-tagged Leu1, Δ34
pET-Duet1	N-terminally His-tagged *E. coli* LeuB

Data S1.

Tabulation of C-terminal proteomes of Fe-S proteins in *H. sapiens, S. cerevisiae, A thaliana, E. coli,* and *M. jannaschii*. Summary of the prokaryotic and archaeal origins.

Data S2.

Analysis of C-terminal tails of CIA clients, factors, and adaptors from 10 eukaryotic reference organisms.

References for SI

1. L. Zheng, V. L. Cash, D. H. Flint, D. R. Dean, Assembly of iron-sulfur clusters. Identification of an *iscSUA-hscBA-fdx* gene cluster from *Azotobacter vinelandii*. *J. Biol. Chem.* **273**, 13264-13272 (1998).
2. K. Stegmaier *et al.*, Apd1 and Aim32 are prototypes of bis-histidinyl-coordinated non-Rieske [2Fe-2S] proteins. *J. Am. Chem. Soc.* **114**, 5753-5765 (2019).
3. A. Vo *et al.*, Identifying the Protein Interactions of the Cytosolic Iron-Sulfur Cluster Targeting Complex Essential for Its Assembly and Recognition of Apo-Targets. *Biochemistry* **57**, 2349-2358 (2018).
4. A. T. Vo *et al.*, Defining the domains of Cia2 required for its essential function in vivo and in vitro. *Metallomics* **9**, 1645-1654 (2017).
5. C. Janke *et al.*, A versatile toolbox for PCR-based tagging of yeast genes: new fluorescent proteins, more markers and promoter substitution cassettes. *Yeast* **21**, 947-962 (2004).
6. L. Zheng, U. Baumann, J. L. Reymond, An efficient one-step site-directed and site-saturation mutagenesis protocol. *Nucleic Acids Res.* **32**, e115 (2004).
7. D. Mumberg, R. Müller, M. Funk, Regulatable promoters of *Saccharomyces cerevisiae*: comparison of transcriptional activity and their use for heterologous expression. *Nucleic Acids Res.* **22**, 5767-5768 (1994).
8. A. V. Bryksin, I. Matsumura, Overlap extension PCR cloning: a simple and reliable way to create recombinant plasmids. *Biotechniques* **48**, 463-465 (2010).
9. A. J. Pierik, D. J. Netz, R. Lill, Analysis of iron-sulfur protein maturation in eukaryotes. *Nat. Protoc.* **4**, 753-766 (2009).
10. A. Bensadoun, D. Weinstein, Assay of proteins in the presence of interfering materials. *Anal. Biochem.* **70**, 241-250 (1976).
11. J. Goa, A micro biuret method for protein determination; determination of total protein in cerebrospinal fluid. *Scand. J. Clin. Lab. Invest.* **5**, 218-222 (1953).
12. F. Madeira *et al.*, Search and sequence analysis tools services from EMBL-EBI in 2022. *Nucleic Acids Res.* **50**, W276-W279 (2022).
13. C. Andreini, L. Banci, A. Rosato, Exploiting Bacterial Operons To Illuminate Human Iron-Sulfur Proteins. *J. Proteome Res.* **15**, 1308-1322 (2016).
14. D. J. A. Netz, J. Mascarenhas, O. Stehling, A. J. Pierik, R. Lill, Maturation of cytosolic and nuclear iron-sulfur proteins. *Trends Cell. Biol.* **24**, 303-312 (2014).
15. M. Lénon, R. Arias-Cartín, F. Barras, The Fe-S proteome of *Escherichia coli*: prediction, function, and fate. *Metallomics* **14** (2022).
16. C. Andreini, A. Rosato, L. Banci, The Relationship between Environmental Dioxygen and Iron-Sulfur Proteins Explored at the Genome Level. *PLoS One* **12**, e0171279 (2017).
17. J. Przybyla-Toscano, C. Boussardon, S. R. Law, N. Rouhier, O. Keech, Gene atlas of iron-containing proteins in *Arabidopsis thaliana*. *Plant J.* **106**, 258-274 (2021).
18. G. E. Crooks, G. Hon, J. M. Chandonia, S. E. Brenner, WebLogo: a sequence logo generator. *Genome Res* **14**, 1188-1190 (2004).
19. D. Kuznetsov *et al.*, OrthoDB v11: annotation of orthologs in the widest sampling of organismal diversity. *Nucleic Acids Res.* **51**, D445-D451 (2023).
20. F. Benisch, E. Boles, The bacterial Entner-Doudoroff pathway does not replace glycolysis in *Saccharomyces cerevisiae* due to the lack of activity of iron-sulfur cluster enzyme 6-phosphogluconate dehydratase. *J. Biotechnol.* **171**, 45-55 (2014).
21. N. Milne, S. A. Wahl, A. J. A. van Maris, J. T. Pronk, J. M. Daran, Excessive by-product formation: A key contributor to low isobutanol yields of engineered *Saccharomyces cerevisiae* strains. *Metab. Eng. Commun.* **3**, 39-51 (2016).
22. F. V. Gambacorta *et al.*, Comparative functional genomics identifies an iron-limited bottleneck in a *Saccharomyces cerevisiae* strain with a cytosolic-localized isobutanol pathway. *Synth. Syst. Biotechnol.* **7**, 738-749 (2022).
23. L. Salusjärvi *et al.*, Production of ethylene glycol or glycolic acid from D-xylose in *Saccharomyces cerevisiae*. *Appl. Microbiol. Biotechnol.* **101**, 8151-8163 (2017).

24. L. Wasserstrom *et al.*, Exploring D-xylose oxidation in *Saccharomyces cerevisiae* through the Weimberg pathway. *AMB Express* **8**, 33 (2018).
25. S. Partow, V. Siewers, L. Daviet, M. Schalk, J. Nielsen, Reconstruction and evaluation of the synthetic bacterial MEP pathway in *Saccharomyces cerevisiae*. *PLoS One* **7**, e52498 (2012).
26. S. Carlsen *et al.*, Heterologous expression and characterization of bacterial 2-C-methyl-D-erythritol-4-phosphate pathway in *Saccharomyces cerevisiae*. *Appl. Microbiol. Biotechnol.* **97**, 5753-5769 (2013).
27. J. Kirby *et al.*, Engineering a functional 1-deoxy-D-xylulose 5-phosphate (DXP) pathway in *Saccharomyces cerevisiae*. *Metab. Eng. Commun.* **38**, 494-503 (2016).
28. F. Desiere *et al.*, The PeptideAtlas project. *Nucleic Acids Res.* **34**, D655-D658 (2006).
29. V. D. Paul *et al.*, The deca-GX_3 proteins Yae1-Lto1 function as adaptors recruiting the ABC protein Rli1 for iron-sulfur cluster insertion. *Elife* **4**, e08231 (2015).
30. U. Mühlenhoff *et al.*, Functional characterization of the eukaryotic cysteine desulfurase Nfs1p from *Saccharomyces cerevisiae*. *J. Biol. Chem.* **279**, 36906-36915 (2004).
31. D. J. Netz, A. J. Pierik, M. Stümpfig, U. Muhlenhoff, R. Lill, The Cfd1-Nbp35 complex acts as a scaffold for iron-sulfur protein assembly in the yeast cytosol. *Nat. Chem. Biol.* **3**, 278-286 (2007).
32. J. Balk, D. J. A. Netz, K. Tepper, A. J. Pierik, R. Lill, The essential WD40 protein Cia1 is involved in a late step of cytosolic and nuclear iron-sulfur protein assembly. *Mol. Cell. Biol.* **25**, 10833-10841 (2005).
33. J. Balk, A. J. Pierik, D. J. Netz, U. Mühlenhoff, R. Lill, The hydrogenase-like Nar1p is essential for maturation of cytosolic and nuclear iron-sulphur proteins. *EMBO J.* **23**, 2105-2115 (2004).
